# Desalination of seawater using integrated microbial biofilm/cellulose acetate membrane and silver NPs/activated carbon nanocomposite in a continuous mode

**DOI:** 10.1038/s41598-023-50311-0

**Published:** 2024-01-02

**Authors:** Ebtesam El Bestawy, Adel Salah Abd El-Hameed, Eman Fadl

**Affiliations:** 1 Department of Environmental Studies, Institute of Graduate Studies and Research, Alexandria University, 163 Horria Ave. El-Shatby, P.O. Box 832, Alexandria, Egypt; 2 Department of Materials Science, Institute of Graduate Studies and Research, Alexandria University, 163 Horria Ave. El-Shatby, P.O. Box 832, Alexandria, Egypt

**Keywords:** Environmental biotechnology, Biotechnology

## Abstract

The main objective of the present study was to desalinate seawater using *Bacillus cereus* gravel biofilm and cellulose acetate (CA) membranes with and without silver nanoparticles (AgNPs) as a potent and safe disinfectant for the treated water. Six desalination trials (I, II, III, IV, V and VI) were performed using the proposed biofilm/cellulose membrane. Results confirmed that *Bacillus cereus* gravel biofilm (microbial desalination) is the optimal system for desalination of seawater. It could achieve 45.0% RE (initial salinity: 44,478 mg/L), after only 3 h compared to the other tested treatments. It could also achieve 42, 42, 57, 43 and 59% RE for TDS, EC, TSS, COD and BOD, respectively. To overcome the problem of the residual salinity and reach complete elimination of salt content for potential reuse, multiple units of the proposed biofilm can be used in sequence. As a general conclusion, the *Bacillus cereus* biofilm system can be considered as remarkably efficient, feasible, rapid, clean, renewable, durable, environmentally friendly and easily applied technology compared to the very costly and complicated common desalination technologies. Up to our knowledge, this is the first time microbial biofilm was developed and used as an effective system for seawater desalination.

## Introduction

Water, the most valuable resource for life on the Earth, becomes extremely rare and be destroyed on a global scale due to population growth, expansion of industrial and agricultural activities, promotion of the living standards and climate change^1^. Water covers about 70% of the surface of globe, of which 97% is saline and inappropriate for human consumption as well as industrial and agricultural uses, 2% polar ice caps and only 1% is available as freshwater in rivers, fresh lakes, and ground water, which is suitable for human uses^2,3^. Available freshwater resources are increasingly drained at an alarming rate in many places worldwide^4^. Therefore, there is an urgent necessity for rising freshwater resources through effective and sustainable technologies to treat and manipulate untraditional sources of water (i.e. seawater, groundwater and wastewater)^5^.

Desalination has arisen as an important source of freshwater, on which many countries are currently depending on to get their freshwater demands. In the Middle East arid countries (i.e. Saudi Arabia, United Arab Emirates, and Kuwait), seawater desalination is a vital, dependable and a key method of securing freshwater resource. It is expected to grow in many other countries in the Middle East such as Egypt^6^. The World Health Organization (WHO) states that the permissible limit of salinity in water is 500 mg/L and for special cases up to 1000 mg/L^7^. Many seawater desalination techniques have been developed during the last years, but they are costly (demanding power and/or materials), therefore, they are restricted by many countries, despite being a very important water resource for many countries^8^. A significant amount of research and development has been executed to improve desalination technologies and reduce its high capital and energy expenses^9,10^. It was estimated that 8.78 million tons of oil are needed annually to generate 1.0 million m^3^ of freshwater per day^11^. Finding adequate alternative energy sources for the desalination systems is a very important.

Traditional desalination technologies can be split into three basic categories: (i) thermally-activated systems, where the main processes for separating salts from water are evaporation and condensation^12^, (ii) systems based on membranes that drive salty water through a membrane by applying either pressure or an electric field, leaving salts behind. and (iii) chemically-activated desalination methods. Multi-stage flash (MSF)^13^, multiple-effect (MED)^14,15^, mechanical vapor compression distillation (MVC)^16^, humidification–dehumidification (HDH)^17^, solar (SD)^18,19^, and freezing (FD)^20^ are processes belong to the thermally activated desalination systems. Reverse osmosis (RO)^21^, forward osmosis (FO)^22^ electro-dialysis (ED)^23^ and Nano filtration (NF)^24^ are desalination membrane technologies. Ion-exchange^25^, liquid–liquid extraction^26^ and gas hydrate^27^, or other precipitation schemes are chemically-activated desalination systems that rely on chemical differences rather than pressure differences or phase shift, in contrast to other desalination methods^28^. Such physicochemical technologies are either expensive, energy-intensive and unfriendly to the environment^29^.

As a result, the necessity to develop biological treatment technologies that would lower salinity in an affordable and environmentally responsible way becomes urgent. Such methods avoid the addition of chemicals and use natural proper microorganisms with high capability to lower the amount of TDS in high saline water^30^. Biofilm based treatment technologies have many advantages and clear benefits over traditional approaches, including ease of phase separation, no washout of bacteria and less sensitivity to environmental conditions, among others. Since biofilm based water and wastewater treatment plants are proportionally increased and are among the most effective technologies, a lot of operational studies are focusing on their performance under several conditions^31,32^. Modification of microorganisms with metals nano-particles (NPs) is one of the most attractive and promising methods that use microbial/NPs assemblage to desalinate water^33^. In addition to the different applications of nanotechnology, desalination of water appears to be another fascinating and potential use of nanotechnology in water purification^34^. Among metals, Nano silver has been developed for numerous applications such as textiles, home water purification systems, medical equipment, cosmetics, electronics, and household appliances as an antibacterial agent^35^. Biosynthesis of metal nanoparticles such as using microbes and/or plants has gained a considerable lot of attention as an environmentally friendly emerging approach. Although various strategies were employed for the synthesis of silver nanoparticles, biological reduction is the most preferred way as it offers one step, eco-friendly way. In comparison to microorganisms, plants have been discovered to reduce metal ions at a rate that is substantially faster, and stable metal nanoparticle production has also been confirmed. It is possible to regulate and control the size and structure of the nanoparticles made from plants by altering the pH. The antibacterial activity of the silver nanoparticles (AgNPs), synthesized using olive leaf extracts (OLE), as a reducing and stabilizing agent, against several bacterial isolates was reported and tested. It is well known that Ag ions and Ag-based compounds have severe biocidal effects on bacteria, fungi, and viruses^36^. Moreover, the olive plant has always been a valuable source of nutrition and medicine^37,38^. Also, AgNPs are more stable, cost-effective, have a regulated release rate, possess great antagonistic impact on microorganisms. Therefore, they have remarkable potential use in the disinfection water and wastewater as well as removal of inorganic anions, heavy metals and organic contaminants.

AgNPs modified cellulose fibers filter may be a superior option to reduce the aggregation issue and provide effective antibacterial potential against (i.e. *E. coli*) at a reasonable price^39^. Composites made of Ag NPs are even better and able to perform multiple tasks since they can both kill certain species and destroy their moieties in a single treatment^40^. The present study aimed to desalinate seawater using the proposed *Bacillus cereus* gravel biofilm filter (as a novel approach) compared with cellulose acetate (CA) membrane filter (as a traditional approach). AgNPs were manipulated to modify cellulose membranes as a potent and safe disinfectant for the treated water.

Up to our knowledge, this is the first time microbial biofilm was developed and used as an effective system for seawater desalination. It is a novel approach, which is remarkably efficient, feasible, rapid, clean, renewable, durable, environmentally friendly and easily applied technology compared to the very costly and complicated common desalination technologies.

## Materials and methods

### Materials

Olive leaves were collected from local farm, Borg El-Arab City, Alexandria and activated carbon was supplied from local market. Ammonium hydroxide solution (NH_4_OH), silver nitrate (AgNO_3_) and extra pure hydrochloric acid (HCL) were purchased from Merck (Darmstadt, Germany). Ethanol and nutrient broth were purchased from Analar and Oxoid, England, respectively. Acetone (purity 99%) was received from Chem Lab, France, cellulose acetate powder (MW. 100,000) from Acros, USA, 1,4 Dioxane from Merck, USA. Acetic acid and methanol were received from Fisher scientific, U.K. All the chemicals were used without any modification.

### Methods

#### Sampling

High salinity water samples were collected from four selected representative sites, namely Abu Qir (east), Gleem, EL-Selsela (central) and El-Anfoushi (west) along the coastal strip of the Mediterranean Sea of Alexandria Governorate, Egypt. Also, other water samples were collected from the red salt marshes at Wadi El-Qamar costal road and from El-Mex Salts Production Company, south Alexandria Governorate, Egypt during the course of the study. Water samples were subjected to physicochemical as well as microbiological characterization to define their pollution strength and select the best treatment technology. Moreover, post-treatment characterization took place to calculate the treatment efficiency and evaluate its suitability.

#### Microorganisms

*Bacillus cereus,* an exogenous species, considered the most efficient exogenous species when investigated with 8 indigenous bacterial isolates from highly saline waters, for desalination and treatment of raw seawater in batch mode bioassays [batch bioassay]. *Bacillus cereus* was previously isolated and identified from salt marshes, Alexandria^41^. In the present study, *Bacillus cereus* was selected, decorated with silver NPs/Activated Carbon Nanocomposite (AgNPs/AC-NC), fixed as a biofilm and investigated in a continuous mode with and without cellulose acetate membrane (CA) for desalination of raw seawater.

### Green synthesis of AgNPs using olive leaves extracts

#### Preparation of olive leaves extract (OLE)

Olive leaves were separated and rinsed well with distilled water, dried at 25 °C and grinded. An amount of 7.5 g of grinding leaves was weighed, to which 100 mL demineralized water was added, and boiled with stirring for 15 min. The solution was filtered using Whattman filter paper. Filtrate was taken and volume was completed to 250 mL with demineralized water.

#### Preparation of AgNPs/activated carbon (AC) nanocomposite (NC)

AgNPs/AC-NC was prepared by diluting 0.34 mL of 0.02 M silver nitrate (Merck Darmstadt, Germany) in 100 mL demineralized water. Different ratios of 0.02 M AgNO_3_ to OLE solutions (1:1, 1:3, 1:4 and 1:5) were prepared and stirred for 20 min, the color changed from white to dark confirming AgNPs formation. AgNPs solution was amended by 50 g activated carbon, vigorously stirred for 2 h, sonicated at 100 Hz for 40 min and dried in oven at 70 °C to form AgNPs/AC-NC. Due to its high degree of microporosity, 1 g of activated carbon has a surface area in excess of 3000 m^2^ (32,000 sq.ft.). Activated carbons (R 1) are made in particulate form as powders or fine granules less than 1.0 mm in size with an average diameter between 0.15 and 0.25 mm that retained on a 50-mesh sieve (0.297 mm). Thus they present a large surface to volume ratio with a small diffusion distance^42,43^ (Supplementary Figs. S1 and S2).

#### Characterization of AgNPs and AgNPs/AC-NC

Structural and compositional characterization of AgNPs (0.02 M) and Ag/AC-NC were performed using ultra violet-visible absorption (UV–VIS), Fourier transform infrared spectrophotometer (FTIR), X ray diffraction (XRD), scanning electron microscope (SEM) and transmission electron microscope (TEM).

#### Ultraviolet–visible (UV–VIS) absorption

Ultraviolet–visible (UV–VIS) absorption spectrum of AgNPs solution was determined using UV–VIS spectrophotometer (Evolution 300 UV–Visible Spectrophotometer, Thermo Scientific, USA) in the range from 300 to 700 nm to determine the characteristics peaks of AgNPs.

#### Fourier transform infrared spectrophotometer (FTIR)

Fourier transform infrared (FTIR) (Spectrum BX 11 Infrared spectrometer LX 18-5255 Perkin Elmer) was used for characterizing the chemical structure of AgNPs and Ag/AC-NC. The spectra were recorded in the wavenumber range of 4000–400 cm^−1^. First, the samples were prepared by grinding with KBr to reduce the particle size. The powder mixture is then pressed in a hydraulic press to form a translucent pellet through which the beam of the spectrometer can pass.

#### X-ray diffraction (XRD)

XRD scan of Ag/AC-NC was determined using X-ray 7000 Shimadzu-Japan at room temperature. The Bragge angle (2ϴ) has the range from 0.9 to 100° to determine the crystallinity of the prepared samples. The X-ray source was Cu target generated at 220 kV and 180 mA with scan speed 2 deg./min.

#### Scanning electron microscope (SEM)

The morphology of the prepared Ag/AC-NC was analyzed using scanning electron microscopy (SEM “JEOL JSM-5300”). Sample was used in powder form and was coated with gold layer to increase the conductivity.

#### High-resolution transmission electron microscope (HR-TEM)

The morphology and particle size of Ag/AC-NC was determined using high resolution transmission electron microscope (HR-TEM) (JEM-1400). TEM sample was prepared by dispersing 2 mg of powder sample in 5 mL of ethanol and sonicated. A drop of this colloidal solution was evaporated on a copper grid and tested.

#### Development of the AgNPs/AC-NC-coated bacterial assemblage

*Bacillus cereus* (the most active and promising strain) was selected and cultured for 24 h at 37 °C in nutrient dehydrated broth (NB) medium (Oxoid, England) till the microbial density reached ∼ 0.5 g [dry weight cells/L]. Ag/AC-NC-(1.5 g) dissolved in 100 mL solution (50 mL DW + 50 mL absolute ethanol) in a conical flask and incubated for 10 min in a sonicator water bath. Ag/AC-NC powder breakage was achieved by sonication shock waves that help breaking intermolecular interactions, thus, speed dissolution. Then, a fixed volume of the culture (200 mL = 0.10 g bacterial weight) was mixed with the prepared Ag/AC-NC solution equivalent to cells: magnetite (g/g) ratio of 1:3 (that resulted in the highest coating efficiency according to El Bestawy et al*.*^44^ using shaking incubator (NEW BRUNSWICK SCIENTIFIC, NEW BRUNSWICK, N.J., USA) at 180 rpm and 37 °C. After 1 h, the Ag/AC-NC-bacterial assemblage was collected and separated from the supernatant plus the free uncoated cells, which can then be harvested.

#### Preparation of CA-RO membranes by phase inversion technique

The CA-RO membrane was prepared using a mixture of acetone (13.5 mL), dioxane (27 g), acetic acid (5 g), methanol (10.7 mL) and cellulose acetate (CA) powder (10.45 g). This mixture was stirred for 24 h at room temperature, until the CA was completely dissolved. The mixture was put into an ultrasonic bath for 30 min to remove the air bubbles entrapped in the polymer solution. The CA-RO membranes were prepared by spreading the solution onto a glass plate using the casting of an automatic applicator (Zehntner ZAA 2300-Swiss), at room temperature and 42% relative humidity. The thickness of the film was adjusted (250 µm).and the spreading was carried out at a constant speed (10 mm/s). After casting, the solvent was evaporated for 30 to 60 s, and the CA membrane (cast onto the glass plate) was immersed for 30 min in a deionized water ice bath. The formed CA membrane was then placed in a water bath at about 4 °C for 2 h and then washed with distilled water to completely remove the residual solvents. The prepared CA-RO membranes were then annealed for (3, 10 min) at about 75 °C. Then membranes were immersed in deionized water for 24 h and air dried for 24 h before characterization^45^. CA-RO membrane with Ag/AC-NC was prepared using phase inversion technique by mixing 0.1 g of Ag/AC-NC with the solvents mixture (in previous step) for 1 h on the stirrer and then repeat the previous procedure of preparation of CA-RO membrane.

#### Characterization of cellulose membrane sheets (CMS)

CMS were characterized for their morphology and elemental analysis using SEM (SEM-JEOL JSM-IT 5300, Japan), for their organic functional groups using FTIR (Model 111653, Liantrisant, UK) and contact angle by measuring the hydrophilicity of the prepared membrane surfaces (Ramé hart, Instrument Company, France) to determine their contact angles. A drop of distilled water (2 μL) was placed on the RO membrane surface (3 × 2 cm) using a micro syringe (Hamilton Company, Reno, NV). The contact angle was calculated as an average of the measurement at 5 different positions within 20 s. after placing the water drop on the membrane surface.

### Development of biofilm system

#### System construction

Supplementary Fig. S3 represents development of the different biofilm treatment systems and their controls. Eight Separating funnels (38.0 × 15.0 × 15.0 cm, 500 mL capacity) were sealed at the bottom by a porous net (d < 1.0 mm) and supplied with a flow controller (tap) at the outlet. They were sterilized by immersing in 75% ethyl alcohol overnight, rinsed twice with absolute ethanol, and five times with sterile distilled water, and then dried in a sterile condition. Gravel aggregates (average particle size 1.4 length × 0.6 width) were used as supporting material after thorough washing, rinsing and sterilization 4 times at 121 °C for 15 min and cellulose membrane sheets (20 cm length × 25 cm width) were also used as supporting material for *B. cereus* biofilm. Two separating funnels were packed with sterile gravel and two separating funnels were packed with sterile gravel and CA membrane sheets. They were filled up to 80% of their height leaving the top 20% free. After packing, one separating funnel of each type was used as a control where only seawater was supplied during the treatment stage, while the other two funnels (with different packing) were inoculated with 400 mL dense overnight *B. cereus* liquid culture and left 14 days to allow bacterial cells adhesion forming the biofilm. One separating funnel was packed with CA membrane sheets modified with Ag/AC-NC, another was packed with unmodified CA membrane sheets (control), both up to 80% of their height leaving the top 20% free. One funnel of the last 2 columns was packed with sterile gravel and CA membrane sheets modified with Ag/AC-NC and inoculated with 400 mL dense overnight *B. cereus* liquid culture while the other funnel was packed with sterile gravel and inoculated with 400 mL dense overnight Ag/AC-NC-modified *B. cereus*. Both funnels were left 14 days to allow bacterial cells adhesion forming the biofilm. The eight separating funnels were connected with an up flow air supply (Fig. S3).

#### Determination of population dynamics

*B. cereus* seeded columns were left as batch cultures for 14 days at pH 7, at room temperature (25–28 °C). A sample from each biofilm column was collected at 24 h interval, serially diluted (up to 10^–8^) and 1000 μL of the appropriate dilution was cultured under aseptic conditions on NA plates and incubated for 24 h at 37 °C. Bacterial plate counts were recorded every day till constant count was obtained for three consecutive days.

#### Operation conditions

Raw seawater samples were initially characterized and then treated using the biofilm and the control filter systems in a continuous mode at different flow rates (200, 400 and 600 mL/h). At each flow rate, samples were collected from both the biofilm and bacteria-free (control) columns at hourly interval for four consecutive hours. After treatment, all samples were characterized for the same parameters and the efficiency of the treatment using the proposed systems were calculated.

#### Characterization of the raw and treated seawater

Quality parameters {pH, temperature, dissolved oxygen content (DO), electrical conductivity (EC), salinity, total suspended solids (TSS), total dissolved solids (TDS), biochemical oxygen demand (BOD), chemical oxygen demand (COD), and total viable count of bacteria (TVC) were determined in the seawater (raw and treated) according to the standard techniques described by Rice et al*.*^46^ in the “Standard Methods for Examination of Water and Wastewater” to determine the removal efficiency at each exposure time.

## Results and discussion

*Bacillus cereus,* was investigated with 8 indigenous bacterial isolates from highly saline waters, for desalination and treatment of raw seawater in batch mode bioassays [batch bioassay]. In that study, the highest RE of the DO, COD, BOD, TDS, Salinity, TSS and EC were achieved by *Bacillus cereus* (81, 66.6, 80.0, 40.0, 42.6, 63.0 and 405%, respectively), reaching RCs (mg/L) of (0.5, 12, 5, 21,000, 27,203, 550 mg/L and 42 ms/cm, respectively) compared to the other tested bacterial isolates. Therefore, it was concluded that *Bacillus cereus* is the most active and the most promising candidate. It was selected to desalinate seawater as fixed biofilm with gravel and/or cellulose membrane sheets as supporting medium for bacterial fixation with and without AgNPs/AC-NC. Six desalination trials (I–VI) were performed using the proposed biofilm and cellulose membrane among which, 3 trials were unmodified and the other 3 were modified with AgNPs/AC-NC.

### Structure analyses

#### Characterization of silver NPs/activated carbon nanocomposite (Ag/AC-NC)

##### Fourier transform infrared spectrophotometer (FTIR)

**Figure 1:**
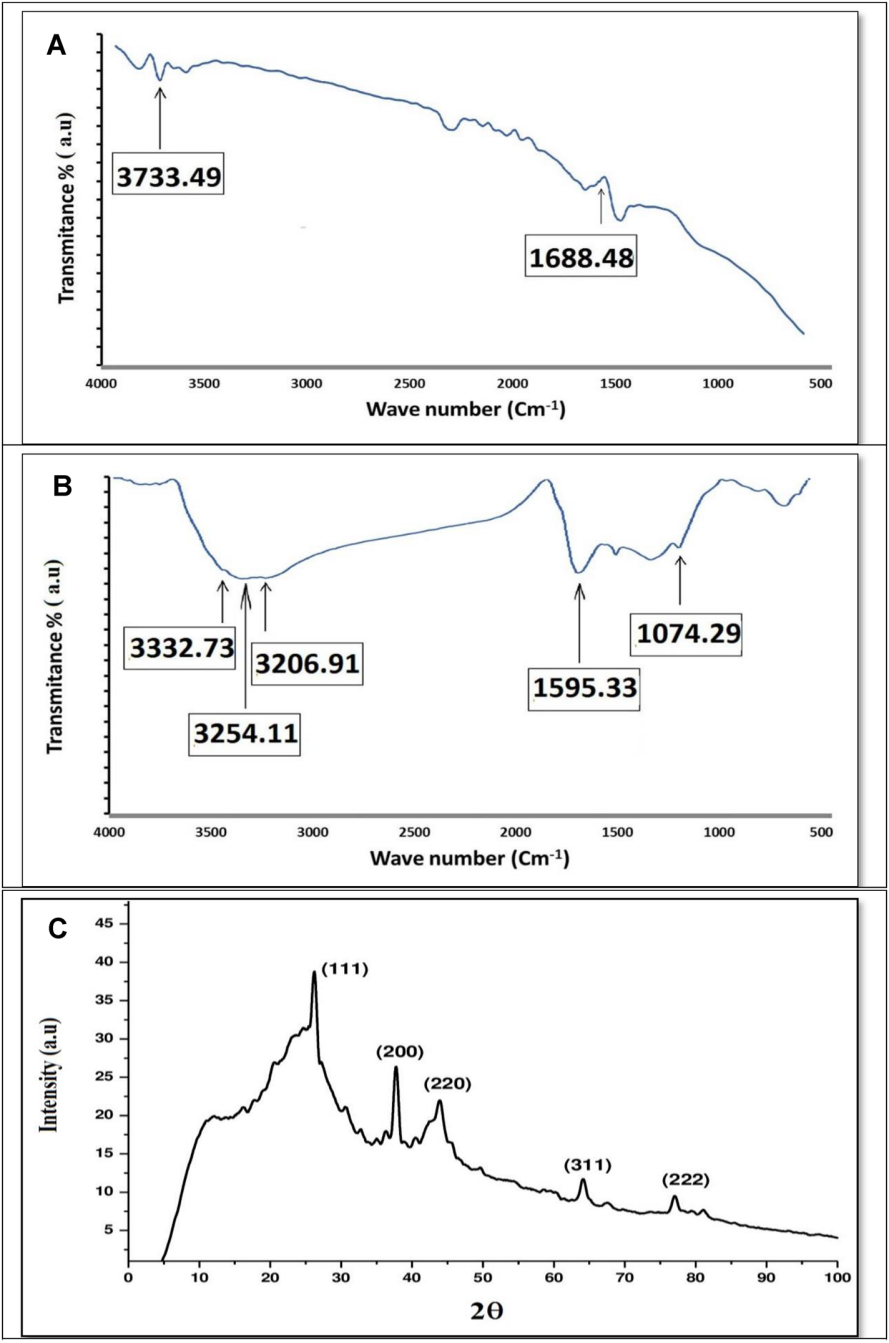
FTIR spectra of AgNPs (**A**), Ag/AC-NC (**B**) and XRD pattern of AgNPs/AC-NC (**C**).

FTIR spectrum of AgNPs and Ag/AC-NC showed a large shift in the absorbance peak with broad band at 3733 cm^−1^ indicated binding of silver ions with hydroxyl groups of the olive leaf extract (Fig. 1A). The band at 1688 cm^−1^ is corresponded to amide I groups that could validate that free amino (–NH_2_) or carboxylate (–COO) groups in the olive leaf extract have interacted with AgNPs surface leading to high stable AgNPs, as explained by Khalil et al*.*^37^. The IR bands at 3332, 3254, and 3206 cm^−1^ (Fig. 1B) are characteristic of the O–H and N–H stretching modes for the OH and amine groups possibly of oleuropein, apigenin-7-glucoside and/or luteolin-7-glucoside present in the olive leaf. The absorption band at 1075 cm^−1^ is characteristic for the C–O stretching bond for alkyl groups. The medium band at 1595 cm^−1^ implying the binding of silver ions with carboxylate groups of the olive leaf extract which have interacted with AgNPs surface making AgNPs highly stable^47^.

##### Crystallinity

XRD pattern of AgNPs/AC-NC (Fig. 1C) illustrates five peaks at 2ϴ around 28, 38, 45, 64 and 78 corresponding to 111, 200, 220, 311 and 222, respectively. The sharpening diffraction peaks indicated the crystalline nature of the synthesized AgNPs. X- Ray data of AgNPs/AC-NC presents crystalline peaks as activated carbon helps in the growth of AgNPs and increase the crystalline structure. The XRD pattern of AgNPs/AC-NC depicts the peaks intensity due to activated carbon coating and shielding the silver nanoparticles. These results are in agreement with Kumar et al. and Sathyavathi et al.^48,49^.

##### Morphological analysis of AgNPs and Ag/AC-NC using scanning and transmission electron microscopy

**Figure 2:**
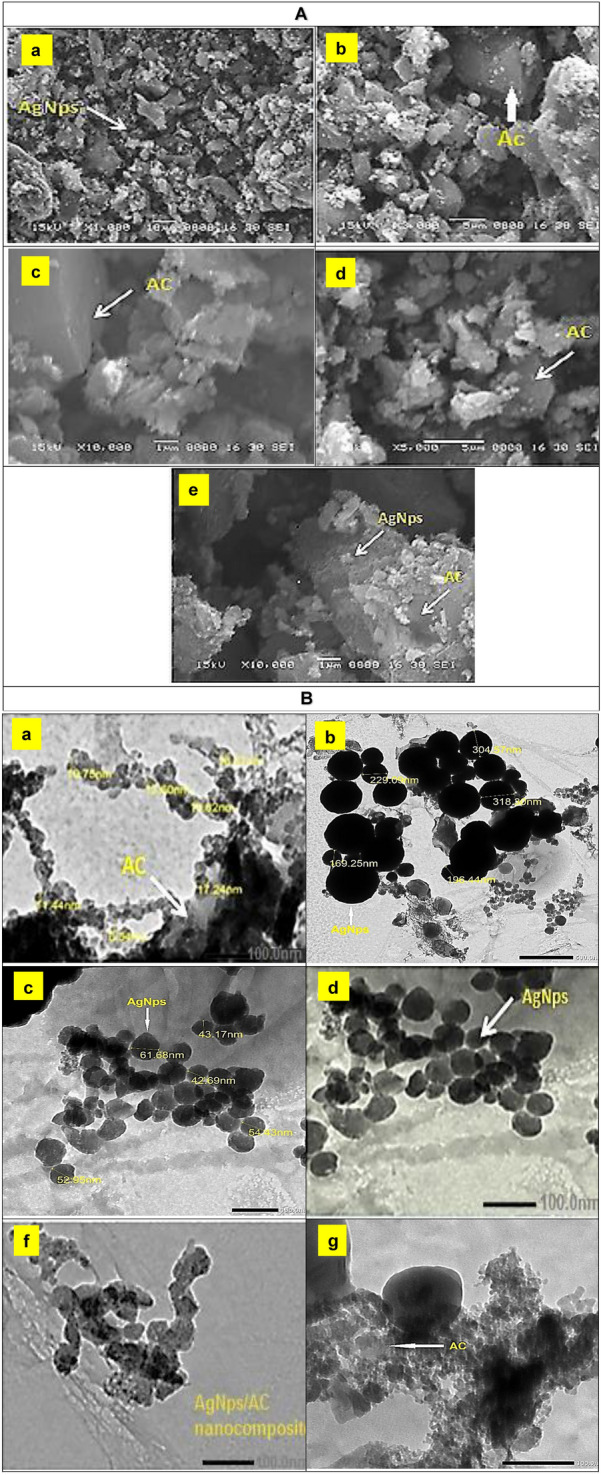
SEM micrographs of AgNPs and AgNPs/AC-NC (**A**) (Scale: a: ×1000, b: ×3000, c: ×10,000, d: ×5000 and e: ×10,000) and TEM Micrographs (**B**) of Synthesized AgNPs and AgNPs/AC-NC (Scale: a: 100 nm, b: 500 nm, c: 100 nm, d: 100 nm, f: 100 nm and g: 100 nm).

The morphology of AgNPs/AC-NC was investigated using SEM (Fig. 2A). The image illustrated the formation of agglomerated AgNPs coated on the surface of activated carbon. The morphology of the nanoparticles was found to be spherical, monodispersed and uniformly distributed. AgNPs in the nanocomposite were recognized as bright dots, which confirms that incorporation process was successful. TEM images of Ag/AC-NC showed that AgNPs are spherical in shape with the presence of some agglomeration and the particle size ranged from 42.69 to 61.68 nm (Fig. 2B).

#### Characterization of cellulose acetate membrane and gravel biofilm

##### Fourier transform infrared spectrophotometer (FTIR)

**Figure 3:**
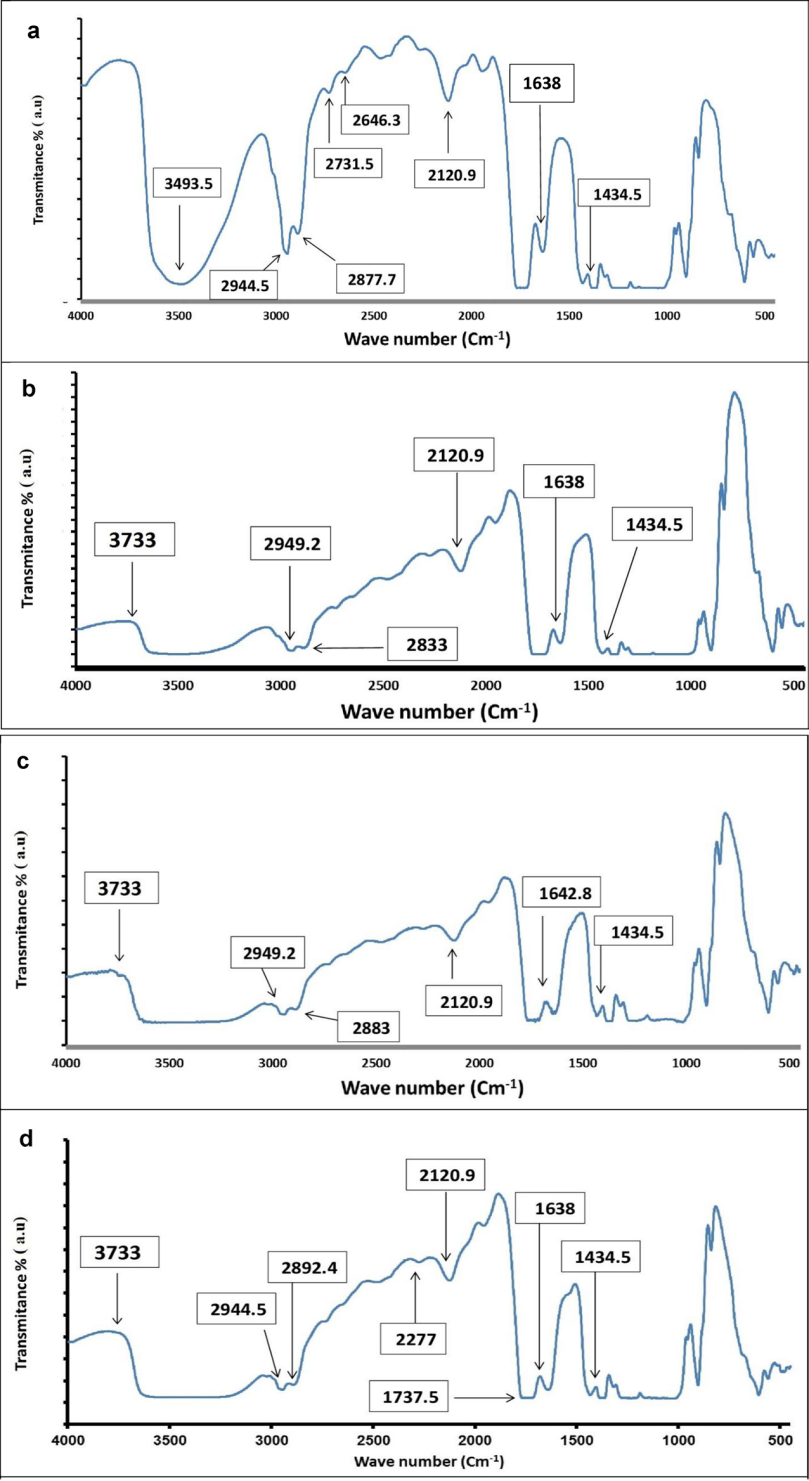
FTIR spectra of pristine CA (**a**), AgNPs nano composite-modified CA (**b**) before treatment, CA/AgNPs nanocomposite (**c**) and AgNPs nano composite-modified CA/*Bacillus cereus* biofilm (**d**), after seawater treatment.

FTIR is a useful tool used for analyzing the chemical structure and the molecular interaction by detecting the specific functional groups in the wave number range of 4000–400 cm^−1^ in the unmodified and modified cellulose acetate membrane. The FTIR spectrum of the pristine cellulose acetate membrane showed a broad band at 3490 cm^−1^ which is assigned to the stretching vibration of the O–H groups. The peaks at 2944, 2887 and 2731 cm^−1^ indicate C–H stretching, peak at 2120 cm^−1^ indicates C≡C stretching and peak at 1060 cm^−1^ indicates C–O stretching. Another fingerprint peak at 1638 cm^−1^ is attributed to CH_2_ symmetric stretching reflects the presence of a carbonyl group (C=O) in cellulose acetate membrane (Fig. 3a). The FTIR spectrum of AgNPs Nano composite-modified CA (before treatment) showed a large shift in the absorbance peak with broad band at 3733 cm^−1^ indicated binding of silver ions with hydroxyl groups of the olive leaf extract. The peaks 2950 and 2883 cm^−1^ indicate C–H stretching. Peaks at 2120 cm^−1^ 1060 cm^−1^ indicated C≡C stretching and C–O stretching, respectively. Another fingerprint peak at around 1434 cm^−1^ indicating CH_2_ bending. Moreover, the peak at 1638 cm^−1^ is attributed to CH_2_ symmetric stretching reflects the presence of a carbonyl group (C=O) in cellulose acetate membrane (Fig. 3b).

After seawater treatment, the FTIR spectrum of AgNPs Nano composite-modified CA showed a large shift in the absorbance peak with broad band at 3733 cm^−1^ indicated binding of silver ions with hydroxyl groups of the olive leaf extract. The peaks 2949 and 2883 cm^−1^ indicated C–H stretching while peaks at 2120 cm^−1^ and 1060 cm^−1^ indicated C≡C and C–O stretching, respectively. Another fingerprint peak at around 1434 cm^−1^ indicated CH_2_ bending. Moreover, the peak at 1642 cm^−1^ is attributed to CH_2_ symmetric stretching, which reflected the presence of a carbonyl group (C=O) in cellulose acetate membrane (Fig. 3c). After seawater treatment, FTIR spectra of AgNPs Nano composite-modified CA and bacterial biofilm showed large shift in the absorbance peaks with broad band at 3733 cm^−1^ indicated binding of silver ions with hydroxyl groups of the olive leaf extract. The peaks 2944 and 2852 cm^−1^ indicated C–H stretching, peak at 2277 cm^−1^ indicated C≡N stretching, peak at 2120 cm^−1^ indicated C≡C stretching and that at 1060 cm^−1^ indicated C–O stretching. Another fingerprint peak at around 1434 cm^−1^ indicated CH_2_ bending. Moreover, the peak at 1737 cm^−1^ is attributed to CH_2_ symmetric stretching reflected the presence of a carbonyl group (C=O) in cellulose acetate membrane (Fig. 3d). These results are consistent with other researchers dealing with CA membranes^47^.

##### Scanning electron microscopy (SEM)

**Figure 4:**
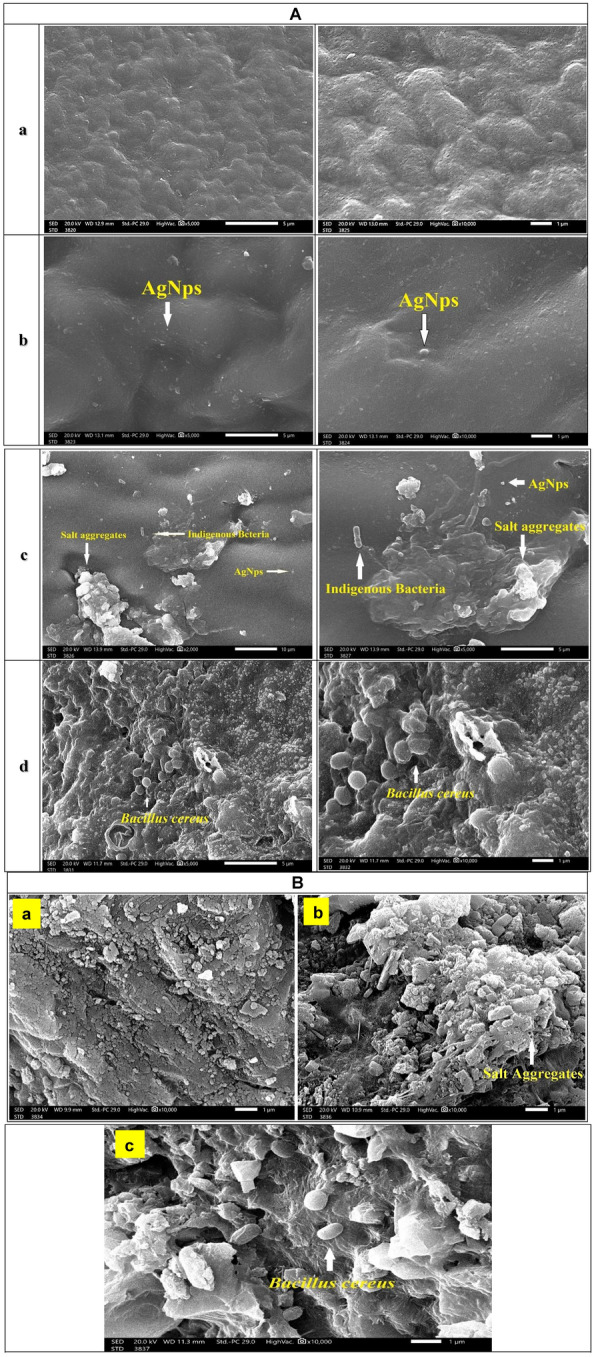
SEM micrographs (**A**) of (a) CA pristine, (b) CA/AgNPs nanocomposite, (c) CA/AgNPs nanocomposite after seawater treatment and (d) CA/AgNPs nanocomposite as well as (**B**) SEM micrographs of (a) gravel particles before treatment, gravel particles with salt aggregates (b) and (c) *Bacillus cereus* gravel biofilm after seawater treatment.

SEM micrograph illustrated the surface morphology of the unmodified cellulose acetate membrane before treatment (Fig. 4A-a) and AgNPs modified cellulose acetate membrane before treatment with the presence of AgNPs on the membrane surface (Fig. 4A-b). After desalination of seawater, SEM micrograph illustrated the presence of AgNPs and salt aggregates as well as some seawater indigenous bacteria on the surface of cellulose acetate membrane (Fig. 4A-c). Moreover, after the desalination process, Fig. 4A-d showed the surface morphology of AgNPs modified cellulose membrane with *Bacillus cereus* fixed biofilm, as well as the presence of AgNPs, salt aggregates and *Bacillus cereus* scattered on the membrane surface. Figure 4B represents SEM micrographs of the gravel particles surface morphology before treatment (Fig. 4B-a) and gravel particles after seawater treatment with salt aggregates and *Bacillus cereus* scattered on the gravel surface (Fig. 4B-b and c).

##### Hydrophilic properties of the pristine and AgNPs-NC modified CA

**Figure 5:**
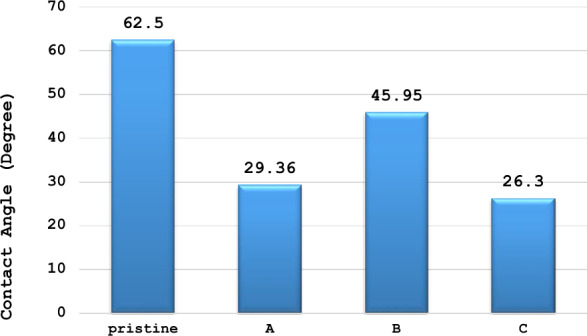
Contact angle of the pristine CA, AgNPs-NC modified CA (**A**), AgNPs-NC modified CA (**B**) and AgNPs-NC modified CA with *Bacillus cereus* biofilm (**C**), after seawater treatment.

Hydrophilic surfaces are thought to strongly adsorb a layer of water molecules, while hydrophobic membrane surfaces are less susceptible to adsorption of species like proteins, bacteria, colloids, etc. and consequently are less sensitive to fouling. Figure 5 indicated that pristine CA membrane had a contact angle of 62.5°, while AgNPs-NC modified CA (A) have less contact angle equal to 29.36° due to the presence of AgNPs on the membrane surface making the membrane negatively charged and more hydrophilic. After seawater treatment, AgNPs-NC modified CA showed an increase in the contact angle equal to 45.95° (B) due to fouling. AgNPs-NC modified CA coupled with bacterial biofilm showed a decrease in the contact angle recording 26.3° (C), due to the presence of *Bacillus cereus,* which makes a protective layer on the surface of the membrane. The enhanced hydrophilic property is attributed to the presence of the functional hydrophilic groups present in the AgNPs.

The contact angle measurements provide compelling evidences that AgNPs are present on the membrane surface. Therefore, it is shown that the integration of AgNPs into the cellulose acetate membranes results in membranes with modified surface properties. Thus, it can be concluded that the unique properties of AgNPs can be used during water separation.

### Continuous desalination of seawater using unmodified biofilm and cellulose membrane

#### Trial I: Seawater desalination using *Bacilluscereus* gravel biofilm system

**Figure 6:**
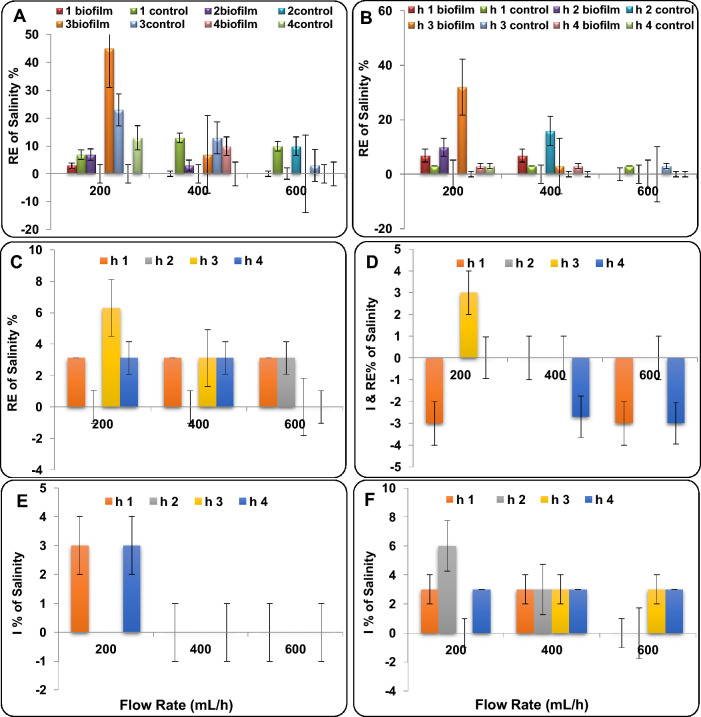
Removal efficiency/increase (RE/I%) of salinity using *Bacillus cereus* gravel biofilm (**A**), *Bacillus cereus* gravel biofilm and unmodified cellulose membrane filter (**B**), unmodified cellulose membrane sheets filter (**C**), AgNPs/AC-NC/gravel biofilm (**D**), gravel biofilm/AgNPs/AC-NC modified cellulose membrane filter (**E**), AgNPs/AC-NC modified cellulose membrane/gravel filter (**F**) systems, at different flow rates and running time.

Population dynamics of *Bacillus cereus* fixed on gravel was determined and results indicated that 12 days were required for the maturation of the proposed biofilm. After maturation, *B. cereus* gravel biofilm system was investigated in a continuous treatment at the different flow rates (200, 400 and 600 mL/h) for 4 h (Supplementary Table S1). Results revealed the following pionts:

1. Raw seawater contained low DO (1.5 mg/L) and organic load (COD and BOD: 40 and 22 mg/L, respectively), high level of TSS (1100 mg/L) and very high levels of TDS, EC and salinity (33,000 mg/L, 66 ms/cm and 44,478 mg/L, respectively).
2. The highest increase in the DO level (113%, 3.2 mg/L) was achieved by the proposed biofilm system at the highest flow rate (600 mL/h) after 4 h (Supplementary Fig. S1).
3. Generally, high removal of Salinity (Fig. 6A), TDS and EC were recorded after treatment using the *Bacillus cereus* gravel biofilm reaching the highest of 42, 42 and 45%, (19,000 mg/L, 38 ms/cm and 24,398 mg/L), respectively, all after 3 running hours at 200 mL/h flow rate (Supplementary Figs. S1 and S2).
4. The highest REs of the TSS, COD and BOD (57, 43 and 59%) equivalent to 470, 23 and 9 mg/L, respectively were achieved by the control (TSS), and biofilm (COD and BOD), all at 600 mL/h flow rate after 3, 4 and 4 running hour, respectively (Supplementary Fig. S2).

Biofilm system is a well-developed technology, very common in wastewater treatment systems, in which solid media are used as attachment surfaces for biofilms. They have several advantages including increasing microbial active biomass density, operational flexibility, low space requirements, reduced hydraulic retention time, resilience to changes in the environment, increased biomass residence time, enhanced ability to degrade recalcitrant compounds through biodegradation, bioaccumulation, biosorption and bio-mineralization as well as lower sludge production. The biofilter-treated water is either released to the environment or used for agriculture and other recreational purposes^50^. However, biofilms are not commonly used in desalination purposes, oppositely, biofouling resulted by marine bacteria-forming biofilm always creates problems and challenges in membrane based—desalination processes^51–53^.

Up to the knowledge, this is the first time, microbial biofilm is developed and used as an effective seawater desalination system. *Bacillus cereus,* considered the most efficient for removing the tested parameters from seawater in a complementary unpublished work by the authors. *Bacillus cereus* is a Gram-positive rod-shaped bacterium commonly found in soil, food, and marine sponges^54^. Some strains are harmful to humans and cause foodborne illness due to their spore-forming nature, while other strains can be beneficial as probiotics for animals, and even exhibit mutualism with certain plants^55–57^. Most strains are mesophilic, having an optimal temperature between 25 and 37 °C, and neutrophilic, preferring neutral pH, but some have been found to grow in environments with much more extreme conditions^58^. It is salt lover (halophilic) as confirmed with its high ability to uptake salts in a very short time in the present study and in previous work^41^.

Not only desalination, *Bacillus cereus* proved strong possibilities for its application in ecological bioremediation. Its ability to decrease heavy metal concentrations via bioaccumulation and biotransformation, increasing phosphorus, nitrogen, and potassium uptake in certain plants was reported^57^. Evidence of bioremediation potential by *B. cereus* was also found in the aquatic ecosystem, where organic nitrogen and phosphorus wastes polluting an eutrophic lake were broken down in its presence^59^. Moreover, *B. cereus* exhibited high ability to degrade keratin in chicken feathers, via hydrolytic mechanisms, indicating its potential to degrade keratinous waste from the poultry industry for potential recycling of the byproducts^60^. These remarkable features of *B. cereus* to deal with environmental contaminants were documented during the present study by achieving 42, 42, 57, 43 and 59% RE for TDS, EC, TSS, COD and BOD reaching RCs of 19,000, 38 (ms/cm), 470, 23 and 9 mg/L, respectively.

Strains of *B. cereus* are both spore-forming, biofilm-forming, display flagellar motility that aid in biofilm formation and spread the biofilm over a larger surface area, and to recruit planktonic, or single, free-living bacteria^61^. *B. cereus* stains have a wide range of virulence factors^62,63^, coupled with its remarkable tendency to form biofilm even by their spore forms^64^, aid to overcome any environmental stresses such as the halophilic conditions during desalination of seawater.

#### Trial II: Seawater desalination using *Bacilluscereus* gravel biofilm and unmodified cellulose membrane filter system

1. Raw seawater contained low DO (1.2 mg/L), organic load (COD and BOD: 37 and 21 mg/L, respectively), high level of TSS (1000 mg/L) and very high levels of TDS, EC and salinity (33,000 mg/L, 66 ms/cm and 44,478 mg/L, respectively) (Table S2).
2. The highest increase in the DO level (100%, 2.8 mg/L) was achieved by the proposed biofilm system at the highest flow rate (600 mL/h) after 4 h (Fig. S3).
3. Generally, low decreases in the levels of Salinity (Fig. 6B), TDS, EC and Salinity were recorded after treatment using the *Bacillus cereus* gravel biofilm and cellulose membrane- reaching the highest of 30, 15 and 32%, (23,000 mg/L, 46 ms/cm and 30,033 mg/L), respectively, all after 3 running hours at 200 mL/h flow rate (Figs. S3 and S4).
4. The highest REs of the TSS, COD and BOD (40, 30 and 57%) equivalent to 600, 26 and 9 mg/L, respectively were achieved by the control, biofilm and biofilm systems, all at 600 mL/h flow rate after 4 running hour (Fig. S4).

#### Trial III: Seawater desalination using unmodified cellulose membrane sheets filter system

1. Raw seawater contained low organic load (COD and BOD: 33 and 22 mg/L, respectively) and low DO (2.0 mg/L), high level of TSS (1100 mg/L) and intermediate levels of TDS, EC and salinity (34,000 mg/L, 68 ms/cm and 45,946 mg/L, respectively) (Table S3).
2. The highest increase in the DO level (115%, 4.3 mg/L) was achieved by the proposed system at the highest flow rate (600 mL/h) after 4 h (Fig. S5).
3. Generally, low decreases in the levels of salinity (Fig. 6C) TDS, EC and were recorded after treatment using the unmodified cellulose membrane sheets reaching the highest of 5.8, 5.8 and 6.3%, (32,000 mg/L, 64 ms/cm and 43,014 mg/L), respectively, all at 200 mL/h flow rate (Figs. S5 and S6).
4. The highest REs of the TSS, COD and BOD (60, 36 and 45%) equivalent to 440, 21 and 12 mg/L, respectively were all achieved by the unmodified cellulose membrane sheets at 600 mL/h flow rate after 4 running h (Fig. S6).

### Continuous desalination of seawater using biofilm and cellulose membrane modified by AgNPs/AC-NC

#### Trial IV: Desalination of seawater using AgNPs/AC-NC/gravel biofilm filter system

*Bacillus cereus* doped with AgNPs/AC-NC was used to form biofilm on gravel and investigated for continuous desalination of raw seawater at different flow rates (200, 400 and 600 mL/h) for 4 h (Table S4). Population dynamics indicated that 7 days was required for maturation of the proposed biofilm. Results revealed the following points:

1. Raw seawater contained low organic load (COD and BOD: 30 and 20 mg/L, respectively) and low DO (2.5 mg/L), high level of TSS (1200 mg/L) and intermediate levels of TDS, EC and salinity (36,000 mg/L, 72 ms/cm and 48,789 mg/L, respectively).
2. The highest increase in the DO level was achieved by the proposed system (88%, 4.7 mg/L) at the highest flow rate (600 mL/h) after 4 h (Fig. S7).
3. Generally, very low increases in the levels of TDS, EC and salinity were recorded after treatment using the proposed system reaching the highest of 2.7, 2.7 and 3%, (37,000 mg/L, 74 ms/cm and 50,256 mg/L), respectively, at 200 and 600 mL/h flow rates. However, this proposed system showed also removal of TDS, EC and Salinity by 2.7, 2.7 and 3% (35,000 mg/L, 70 ms/cm and 47,317 mg/L), respectively, at 200 mL/h flow rate (Figs. 6D, S7 and S8).
4. The highest REs of the TSS, COD and BOD (42, 37 and 50%) equivalent to 700, 19 and 10 mg/L, respectively were all achieved by the proposed AgNPs/AC-NC/gravel biofilm treatment system at 600 mL/h flow rate after 4 running h (Fig. S8).

#### Trial V: Desalination of seawater using AgNPs/AC-NC modified cellulose membrane/gravel biofilm filter system

The proposed treatment system composed of *Bacillus cereus* biofilm fixed on gravel and AgNPs/AC-NC modified cellulose membrane (act as supporting medium) was investigated for bioremediation of raw seawater at different exposure times. Population dynamics revealed that 10 days were required for the maturation of the proposed biofilm. Results concluded the following points:

1. Raw seawater contained low organic load (COD and BOD: 28 and 18 mg/L, respectively) and DO (2.4 mg/L), high level of TSS (1300 mg/L) and intermediate levels of TDS, EC and salinity (36,000 mg/L, 72 ms/cm and 48,789 mg/L), respectively (Table S5).
2. The highest increase in the DO level was achieved by the proposed system (92%, 4.6 mg/L) at the highest flow rate (600 mL/h) after 4 h (Fig. S9).
3. Generally, very low increases in the levels of TDS, EC and salinity were recorded after treatment using the proposed system reaching the highest of 2.7, 2.7 and 3%, (37,000 mg/L, 74 ms/cm and 50,256 mg/L), respectively, all at 200 mL/h flow rate after 1 h (Figs. 6E, S9 and S10).
4. The highest REs of the TSS, COD and BOD (42, 50 and 67%) equivalent to 750, 14 and 6 mg/L, respectively were all achieved by the proposed gravel/Ag NPs modified cellulose membrane filter system, at 600 mL/h flow rate after 4 running h (Fig. S10).

#### Trial VI: Desalination of seawater using AgNPs/AC-NC modified cellulose membrane/gravel filter system

1. Raw seawater contained low organic load (COD and BOD: 31 and 19 mg/L, respectively), high level of TSS (1060 mg/L) and intermediate levels of TDS, EC and salinity (36,000 mg/L, 72 ms/cm and 48,789 mg/L), respectively (Table S6).
2. The highest increase in the DO level was achieved by the proposed system (120%, 6.6 mg/L) at the highest flow rate (600 mL/h) after 4 h (Fig. S11).
3. Generally, very low increases in the levels of TDS, EC and salinity were recorded after treatment using the proposed system reaching the highest of 5.5, 5.5 and 6% (38,000 mg/L, 76 ms/cm and 51,856 mg/L), respectively, all at 200 mL/h flow rate after 2 h (Figs. 6F, S11 and S12).
4. The highest REs of the TSS, COD and BOD (35, 45 and 63%) equivalent to 690, 17 and 7 mg/L, respectively were all achieved by the proposed gravel/AgNPs modified cellulose membrane filter system, at 600 mL/h flow rate after 4 running h (Fig. S12).

### Comparison among the different continuous desalination treatment systems with and without AgNPs/AC-NC

Table 1 clearly indicated that among the six desalination trials tested during the present study, the first trial using the proposed *Bacillus cereus* gravel biofilm (microbial desalination) is the optimal system for desalination of seawater. This system could achieve 45.0% RE of the salinity (initial concentration: 44,478 mg/L) reaching RC of 24,398 mg/L after only 3 h compared to the other tested treatments. It could also achieve 42, 42, 57, 43 and 59% RE for TDS, EC, TSS, COD and BOD reaching RCs of 19,000, 38 (ms/cm), 470, 23 and 9 mg/L respectively. Concerning salinity removal, the 2nd proposed treatment using *Bacillus cereus* gravel biofilm and unmodified cellulose acetate membrane was second in order followed by the 3rd proposed treatment using unmodified cellulose acetate membrane sheets with 32.0 and 6.3% RE (30,033 and 43,014 mg/L) respectively. Incorporating AgNPs/AC-NC with the proposed biofilm or cellulose acetate membrane did not show any capacity for salinity removal, oppositely salinity increased in the 3 modified treatment trials with very low (3.0%) salinity removal in the 4th trial only, while no removals were recorded in the 5th and 6th trials at all. This is mainly because AgNPs/AC-NC was incorporated as antimicrobial agent for disinfection of the treated water, not for removing salts. These results proved that microbial desalination using potent species such as *Bacillus cereus* considered highly efficient, feasible, rapid, clean, renewable, environmentally friendly and easy applied technology compared to the very costly and complicated common desalination technologies. It could remove almost half of the salinity load from the raw seawater in a very short time (3 h). For complete elimination of salt content, enhancing the water quality and reuse of the produced water for irrigation or industrial purpose, multiple units of the proposed biofilm can be used in sequence.

**Table 1:** Comparison among the different continuous desalination treatment systems with and without AgNPs/AC-NC.

Parameters		System	Treatment system											
			*Bacillus cereus* gravel biofilm		*Bacillus cereus* gravel biofilm and unmodified cellulose		Unmodified cellulose membrane sheets		AgNPs/AC-NC/gravel biofilm		AgNPs/AC-NC modified cellulose membrane/gavel biofilm		AgNPs/AC-NC modified cellulose membrane/gravel filter system	
			RE/I%	RC	RE/I%	RC	RE/I%	RC	RE/I%	RC	I/RE%	RC	I/RE%	RC
DO	(mg/L)	Biofilm	113 (I)	3.2	100 (I)	2.8	115 (I)	4.3	88.0 (I)	4.7	92.0 (I)	4.6	120.0 (I)	6.6
		Control	74 (I)	2.6	86 (I)	2.6								
TDS		Biofilm	42 (RE)	19,000	30 (RE)	23,000	5.8 (RE)	32,000	2.7 (I)	37,000	2.7 (I)	37,000	5.5 (I)	38,000
		Control	21 (RE)	26,000	15 (RE) 150 ml/h (4 h)	28,000			2.7 (RE)	35,000				
EC (ms/cm)		Biofilm	42 (D)	38	30 (D)	46	5.8 (RE)	64	2.7 (I)	74	2.7 (I)	74	5.5 (I)	76
		Control	21 (D)	52	15 (D)	56			2.7 (RE)	70				
Salinity	(mg/L)	Biofilm	45 (RE)	24,398	32 (RE)	30,033	6.3 (RE)	43,014	3.0 I	50,265	3.0 (I)	50,256	6.0 (I)	51,856
		Control	23 (RE)	34,317	16 (RE)	37,198			3.0 (RE)	47,317				
TSS		Biofilm	36 (RE)	700	27 (RE)	730	60 (RE)	440	42 (RE)	700	42.0 (RE)	750	35.0 (RE)	690
		Control	57 (RE)	470	40 (RE)	600								
COD		Biofilm	43 (RE)	23	30 (RE)	26	36 (RE)	21	37 (RE)	19	50.0 (RE)	14	45.0 (RE)	17
		Control	36 (RE)	26	27 (RE)	27								
BOD		Biofilm	59 (RE)	9	57 (RE)	9	45 (RE)	12	50 (RE)	10	67.0 (RE)	6	63 (RE)	7
		Control	45 (RE)	12	52 (RE)	10								

Among the six desalination treatment systems tested during the present study, results clearly indicated that the proposed *Bacillus cereus* gravel biofilm is the optimal system for desalination of seawater (45.0% RE of 44,478 mg/L initial salinity) after only 3 h compared to the other tested treatments. Although effective in generating high-quality drinking water and the best relevant technology for solving water shortage problems worldwide, RO membranes are costly due to the high energy demand as well as cleaning and replacement of the membrane modules^65–67^. Polyvinylchloride/cellulose acetate (PVC/CA) membranes used for seawater desalination led to increase permeate flux and salt rejection reaching 99.99% at low feed concentration (5120 ppm) and 99.95% for Red Seawater (38,528 ppm) but under high pressure (up to 40 bar), and hence energy consumption^68^. However, their durability, performance and separation efficiency are restricted by the type of membrane materials/additives used in the preparation processes^69,70^. Cellulose nanofibers, cellulose nanocrystals, bacterial Nano cellulose and diverse cellulose derivatives exhibit different properties and structures, but all of them could enhance the porosity, pore size, hydrophilic character and thus ultimately salt-removal capability and water permeability of the membrane^69^. Chemical modification of Nano-cellulose can definitely improve the surface affinity and membranes reactivity for efficient separation of specific contaminants/ions^70^.

NF membranes modified with cellulose nanocrystal (CNC) showed ultra-high permeation flux up to 204 L m^−2^ h^−1^ with salt rejection efficiency of Na_2_SO_4_ (97.7%) > MgSO_4_ (86%) > MgCl_2_ (15.5%) > CaCl_2_ (11%) > NaCl (6.5%). CNC molecules were responsible for the rejection performance of the NF membranes at lower operational pressure at 0.6 MPa, thereby making it to an energy-saving process^31^. NF membrane modified with CNC/silver composite showed 25.4 L m^−2^ h^−1^ bar^−1^ water permeability, 99.1% Na_2_SO_4_ rejection rate as well as outstanding antifouling performance with humic acid’s flux recovery ratio of 92.6% and antibacterial properties of 99.4% reduction in *E. coli* feasibility^71^. Introducing CNCs into NF membranes showed a 60% increase in permeability compared to the unmodified NF, reaching 98.7 and 98.8% salt rejection rate for Na_2_SO_4_ and MgSO_4_, respectively^72^.

In the present study, unexpectedly cellulose acetate (CA) membranes showed much lower ability for desalination compared to the *B. cereus* biofilm (45.0 RE%, 24,398 mg/L). This was shown by 32% salts removal (30,033 mg/L) achieved by *B. cereus* gravel biofilm and the unmodified CA membrane and only 6.3 RE% (43,014 mg/L) achieved by the unmodified CA membrane sheets in the third trial. This is clearly confirmed the critical and highly efficient role played by *B. cereus* biofilm in seawater desalination.

Incorporating AgNPs/AC-NC with the proposed biofilm or cellulose acetate membrane did not show any capacity for salinity removal, oppositely salinity increased in the 3 modified treatment trials with very low (3.0%) salinity removal in the 4th trial only while no removals were recorded in the 5th and 6th trials at all. This is mainly because AgNPs/AC-NC was incorporated as antimicrobial agent for disinfection of the treated water, not for removing salts. It is well known that AgNPs possess powerful antibacterial activities and have demonstrated remarkable potential for use in water and wastewater treatment for disinfection because of their great toxicity to microorganisms^39,40^. This clearly explained the sharp decline of salinity removal in the 4^th^ trial from 45.0 RE% to an increase in the salinity by 3% by AgNPs/AC-NC/gravel biofilm due to inhibition of the biofilm bacteria.

## Conclusion

Among the six continuous desalination treatment trials during the present study with and without AgNPs/AC-NC, results clearly indicated that the first trial using the proposed *Bacillus cereus* gravel biofilm (microbial desalination) is the optimal system for desalination of seawater. As far as known, this is the first time, microbial biofilm is intentionally developed and used as an effective desalination system. This system could achieve 45.0% RE of the salinity (initial concentration: 44,478 mg/L) reaching RC of 24,398 mg/L after only 3 h compared to the other tested treatments. It could also achieve 42, 42, 57, 43 and 59% RE for TDS, EC, TSS, COD and BOD reaching RCs of 19,000, 38 (ms/cm), 470, 23 and 9 mg/L, respectively.

In order to achieve complete elimination of the salt content, enhancing the water quality and reuse of the produced water for irrigation or industrial purposes, multiple units of the proposed biofilm can be used in sequence. Results of the present study clearly confirmed that *Bacillus cereus* biofilm system can be considered as remarkably efficient, feasible, rapid, clean, renewable, durable, environmentally friendly and easy applied technology compared to the very costly and complicated common desalination technologies. Therefore, upscaling and investigating the ability of the proposed *Bacillus cereus* biofilm system for desalination of sea water/TDS minimization of industrial and domestic wastewater is recommended as future work.

### Supplementary Information

Supplementary Tables.

Supplementary Figures.

## Data Availability

All relevant data are included in the paper or its Supplementary Information.
