# Supplementary material for: Desalination of seawater using integrated microbial biofilm/cellulose acetate membrane and silver NPs/activated carbon nanocomposite in a continuous mode
Source: Sci Rep. 2024 Jan 2;14:274. doi: 10.1038/s41598-023-50311-0 (PMC10762133; doi:10.1038/s41598-023-50311-0)
Supplement: Supplementary file 1 — Supplementary Tables. [file 41598_2023_50311_MOESM1_ESM.docx]

Table S1 Residual Levels and Increase/ Removal Efficiency% (I/ RE) of the Tested Parameters in the

Seawater after Continuous Treatment Using *Bacillus cereus*/Gravel Biofilm System.

| Time  (h) | | Temperature ºC | | | pH | | |  | DO | | | | | | | |
| --- | --- | --- | --- | --- | --- | --- | --- | --- | --- | --- | --- | --- | --- | --- | --- | --- |
|  |  | Flow Rate (mL/h) | | | | | | | | | | | | | | |
|  |  | 200 | 400 | 600 | 200 | 400 | 600 |  | 200 | | | 400 | | | 600 | |
|  |  |  |  |  |  |  |  |  | RC | I% ^a^ | | RC | | I% | RC | I% |
| 0 | | Temp.: 26 ºC | | | pH: 8.3 | | |  | DO: 1.5 (mg/L) | | | | | | | |
| 1 | B | 28 | 26.7 | 27.5 | 8.1 | 8.1 | 8.1 |  | 1.8 | | 20* | | 2 | 33* | 2.3 | 53 |
|  | C | 28 | 26.8 | 27.3 | 8.2 | 8.1 | 8.1 |  | 1.6 | | 7 | | 1.8 | 20 | 1.9 | 27 |
| 2 | B | 28 | 26.6 | 27.2 | 8.1 | 8.2 | 8.2 |  | 2.3 | | 53 | | 2.4 | 60 | 2.5 | 67 |
|  | C | 28 | 26.9 | 26.9 | 8.1 | 8.2 | 8.2 |  | 1.9 | | 27 | | 2.1 | 40 | 2.2 | 47 |
| 3 | B | 28 | 26.2 | 26.8 | 8.2 | 8.1 | 8.2 |  | 2.6 | | 74 | | 2.7 | 80 | 2.8 | 87 |
|  | C | 28.1 | 26.5 | 26.8 | 8.1 | 8.1 | 8.2 |  | 2.2 | | 47 | | 2.3 | 53 | 2.4 | 60 |
| 4 | B | 27.5 | 26 | 26.8 | 8.2 | 8.0 | 8.2 |  | 2.9 | | 93** | | 3.0 | 100** | 3.2 | 113** |
|  | C | 27 | 26 | 26.8 | 8.2 | 8.1 | 8.2 |  | 2.6 | | 74 | | 2.5 | 67 | 2.6 | 73 |
|  |  |  | | | | | | | | | | | | | | |
|  | 0 | Raw TDS: 33000 (mg/L) | | | | | |  | Raw EC: 66 (ms/cm) | | | | | | | |
|  |  | 200 | | 400 | | 600 | |  | 200 | | | 400 | | | 600 | |
|  |  | RC | RE% | RC | RE%% | RC | RE%% |  | RC | D%^a^ | | RC | | D%^a^ | RC | D%^a^ |
| 1 | B | 32000 | 3* | 33000 | 0 | 33000 | 0 |  | 64 | | 3 | | 66 | 0 | 66 | 0 |
|  | C | 31000 | 6 | 29000 | 12** | 30000 | 9** |  | 62 | | 6 | | 58 | 12** | 60 | 9** |
| 2 | B | 31000 | 6 | 32000 | 3 | 33000 | 0 |  | 62 | | 6 | | 64 | 3 | 66 | 0 |
|  | C | 33000 | 0 | 33000 | 0 | 30000 | 9** |  | 66 | | 0 | | 66 | 0 | 60 | 9** |
| 3 | B | 19000 | 42** | 31000 | 6 | 33000 | 0 |  | 38 | | 42** | | 62 | 6 | 66 | 0 |
|  | C | 26000 | 21 | 29000 | 12** | 32000 | 3 |  | 52 | | 21 | | 58 | 12** | 64 | 3 |
| 4 | B | 33000 | 0 | 30000 | 9 | 33000 | 0 |  | 66 | | 0 | | 60 | 9 | 66 | 0 |
|  | C | 29000 | 12 | 33000 | 0 | 33000 | 0 |  | 58 | | 12 | | 66 | 0 | 66 | 0 |
|  | | | | | | | | | | | | | | | | |
|  | 0 | Raw Salinity: 44478 (mg/L) | | | | | |  | Raw TSS: 1100 (mg/L) | | | | | | | |
|  |  | 200 | | 400 | | 600 | |  | 200 | | | 400 | | | 600 | |
|  |  | RC | I% | RC | I% | RC | I% |  | RC | RE%% | | RC | | RE%% | RC | RE%% |
| 1 | B | 43014 | 3 | 44478 | 0 | 44478 | 0 |  | 800 | | 27 | | 900 | 18 | 950 | 11 |
|  | C | 41554 | 7 | 38646 | 13** | 40098 | 10** |  | 720 | | 35 | | 645 | 41 | 600 | 45 |
| 2 | B | 41554 | 7 | 43014 | 3 | 44478 | 0 |  | 700 | | 36 | | 720 | 35 | 750 | 32 |
|  | C | 44478 | 0 | 44478 | 0 | 40098 | 10** |  | 650 | | 41 | | 670 | 39 | 520 | 53 |
| 3 | B | 24398 | 45** | 41554 | 7 | 44478 | 0 |  | 725 | | 34 | | 770 | 30 | 740 | 33 |
|  | C | 34317 | 23 | 38646 | 13** | 43014 | 3 |  | 550 | | 50** | | 500 | 55** | 470 | 57** |
| 4 | B | 44478 | 0 | 40098 | 10 | 44478 | 0 |  | 760 | | 31 | | 790 | 28 | 760 | 31 |
|  | C | 38646 | 13 | 44478 | 0 | 44478 | 0 |  | 670 | | 39 | | 630 | 43 | 580 | 47 |
|  | | | | | | | | | | | | | | | | |
|  | 0 | Raw COD: 40 (mg/L) | | | | | |  | Raw BOD: 22 (mg/L) | | | | | | | |
|  |  | 200 | | 400 | | 600 | |  | 200 | | | 400 | | | 600 | |
|  |  | RC | RE | RC | RE | RC | RE% |  | RC | RE% | | RC | | RE% | RC | RE% |
| 1 | B | 35 | 13* | 33 | 18* | 31 | 23* |  | 19 | | 14* | | 17 | 23* | 16 | 27* |
|  | C | 38 | 5 | 35 | 13 | 33 | 18 |  | 20 | | 9 | | 19 | 14 | 18 | 18 |
| 2 | B | 31 | 23 | 30 | 25 | 29 | 28 |  | 16 | | 27 | | 15 | 32 | 14 | 36 |
|  | C | 36 | 10 | 32 | 20 | 30 | 25 |  | 18 | | 18 | | 16 | 27 | 17 | 23 |
| 3 | B | 27 | 33 | 26 | 35 | 27 | 33 |  | 14 | | 36 | | 13 | 41 | 12 | 45 |
|  | C | 33 | 18 | 30 | 25 | 28 | 30 |  | 17 | | 23 | | 15 | 32 | 14 | 36 |
| 4 | B | 25 | 38** | 24 | 40** | 23 | 43** |  | 13 | | 41** | | 12 | 45** | 9 | 59** |
|  | C | 32 | 20 | 27 | 33 | 26 | 35 |  | 16 | | 27 | | 13 | 41 | 12 | 45 |
| B: Biofilm; C: Control; **: The Highest RE/Increase %, *: The Lowest RE/Increase %, D^a^: Decrease % | | | | | | | | | | | | | | | | |

Table S2 Residual Levels and Increase/ Removal Efficiency% (I/ RE) of the Tested Parameters in the Seawater after Continuous Treatment Using *Bacillus cereus* Gravel Biofilm and Unmodified Cellulose Membrane Filter System.

| Time  (h) | | Temperature ºC | | | pH | | |  | DO | | | | | | | |
| --- | --- | --- | --- | --- | --- | --- | --- | --- | --- | --- | --- | --- | --- | --- | --- | --- |
|  |  | Flow Rate (mL/h) | | | | | | | | | | | | | | |
|  |  | 200 | 400 | 600 | 200 | 400 | 600 |  | 200 | | | 400 | | | 600 | |
|  |  |  |  |  |  |  |  |  | RC | I% ^a^ | | RC | | I% | RC | I% |
| 0 | | Temp.: 26 ºC | | | pH: 8.3 | | |  | DO: 1.4 (mg/L) | | | | | | | |
| 1 | B | 26.8 | 27.3 | 26.7 | 8.1 | 8.1 | 8.1 |  | 1.8 | | 29* | | 2 | 43* | 2.1 | 50* |
|  | C | 26.6 | 27.3 | 26.7 | 8.2 | 8.1 | 8.1 |  | 1.6 | | 14 | | 1.8 | 29 | 1.9 | 36 |
| 2 | B | 26.7 | 26.7 | 26.4 | 8.1 | 8.2 | 8.2 |  | 2.0 | | 43 | | 2.2 | 57 | 2.4 | 71 |
|  | C | 26.6 | 26.9 | 26.4 | 8.1 | 8.2 | 8.2 |  | 1.8 | | 29 | | 2.1 | 50 | 2.2 | 57 |
| 3 | B | 26.8 | 26.9 | 26.1 | 8.2 | 8.1 | 8.2 |  | 2.3 | | 64 | | 2.5 | 79 | 2.6 | 86 |
|  | C | 26.9 | 27.0 | 26.2 | 8.1 | 8.1 | 8.2 |  | 2.0 | | 43 | | 2.3 | 64 | 2.5 | 79 |
| 4 | B | 26.4 | 26.4 | 26.4 | 8.0 | 8.0 | 8.2 |  | 2.5 | | 79** | | 2.7 | 93** | 2.8 | 100** |
|  | C | 26.3 | 26.3 | 26.5 | 8.1 | 8.1 | 8.2 |  | 2.2 | | 57 | | 2.4 | 71 | 2.6 | 86 |
|  |  |  | | | | | | | | | | | | | | |
|  | 0 | Raw TDS: 33000 (mg/L) | | | | | |  | Raw EC: 66 (ms/cm) | | | | | | | |
|  |  | 200 | | 400 | | 600 | |  | 200 | | | 400 | | | 600 | |
|  |  | RC | RE% | RC | RE%% | RC | RE%% |  | RC | D%^a^ | | RC | | D%^a^ | RC | D%^a^ |
| 1 | B | 31000 | 6 | 31000 | 6 | 33000 | 0 |  | 62 | | 6 | | 62 | 6 | 66 | 0 |
|  | C | 32000 | 3 | 32000 | 3 | 32000 | 3** |  | 64 | | 3 | | 64 | 3 | 64 | 3** |
| 2 | B | 30000 | 9 | 33000 | 0 | 33000 | 0 |  | 60 | | 9 | | 66 | 0 | 66 | 0 |
|  | C | 33000 | 0 | 28000 | 15** | 33000 | 0 |  | 66 | | 0 | | 56 | 15** | 66 | 0 |
| 3 | B | 23000 | 30** | 32000 | 3 | 33000 | 0 |  | 46 | | 30** | | 64 | 3 | 66 | 0 |
|  | C | 33000 | 0 | 33000 | 0 | 32000 | 3 |  | 66 | | 0 | | 66 | 0 | 64 | 3 |
| 4 | B | 32000 | 3* | 32000 | 3* | 33000 | 0* |  | 64 | | 3 | | 64 | 3 | 66 | 0 |
|  | C | 32000 | 3 | 32000 | 3 | 33000 | 0 |  | 64 | | 3 | | 66 | 0 | 66 | 0 |
|  | | | | | | | | | | | | | | | | |
|  | 0 | Raw Salinity: 44478 (mg/L) | | | | | |  | Raw TSS: 1000 (mg/L) | | | | | | | |
|  |  | 200 | | 400 | | 600 | |  | 200 | | | 400 | | | 600 | |
|  |  | RC | I% | RC | I% | RC | I% |  | RC | RE%% | | RC | | RE%% | RC | RE%% |
| 1 | B | 41554 | 7 | 41554 | 7 | 44478 | 0 |  | 900 | | 10 | | 930 | 7 | 950 | 5 |
|  | C | 43014 | 3 | 43014 | 3 | 43014 | 3** |  | 870 | | 13 | | 820 | 18 | 780 | 22 |
| 2 | B | 40098 | 10 | 44478 | 0 | 44478 | 0 |  | 820 | | 18 | | 850 | 15 | 900 | 10 |
|  | C | 44478 | 0 | 37198 | 16** | 44478 | 0 |  | 810 | | 19 | | 790 | 21 | 770 | 23 |
| 3 | B | 30033 | 32** | 43014 | 3 | 44478 | 0 |  | 750 | | 25** | | 770 | 23 | 850 | 15 |
|  | C | 44478 | 0 | 44478 | 0 | 43014 | 3 |  | 730 | | 27 | | 650 | 35** | 600 | 40** |
| 4 | B | 43014 | 3 | 43014 | 3 | 44478 | 0 |  | 770 | | 23 | | 800 | 20 | 730 | 27 |
|  | C | 43014 | 3 | 44478 | 0 | 44478 | 0 |  | 750 | | 25** | | 700 | 30 | 670 | 33 |
|  | | | | | | | | | | | | | | | | |
|  | 0 | Raw COD: 37 (mg/L) | | | | | |  | Raw BOD: 21 (mg/L) | | | | | | | |
|  |  | 200 | | 400 | | 600 | |  | 200 | | | 400 | | | 600 | |
|  |  | RC | RE | RC | RE | RC | RE% |  | RC | RE% | | RC | | RE% | RC | RE% |
| 1 | B | 35 | 5 | 33 | 11 | 32 | 14 |  | 19 | | 10 | | 17 | 19 | 15 | 29 |
|  | C | 36 | 3 | 34 | 8 | 33 | 11 |  | 20 | | 5 | | 18 | 14 | 16 | 24 |
| 2 | B | 33 | 11 | 31 | 16 | 30 | 19 |  | 17 | | 19 | | 15 | 29 | 13 | 38 |
|  | C | 35 | 5 | 32 | 14 | 31 | 16 |  | 18 | | 14 | | 16 | 24 | 14 | 33 |
| 3 | B | 31 | 16 | 29 | 22 | 28 | 24 |  | 14 | | 33 | | 13 | 38 | 11 | 48 |
|  | C | 33 | 11 | 30 | 19 | 29 | 22 |  | 16 | | 24 | | 14 | 33 | 12 | 43 |
| 4 | B | 30 | 19** | 27 | 27** | 26 | 30** |  | 11 | | 48** | | 10 | 52** | 9 | 57** |
|  | C | 31 | 16 | 28 | 24 | 27 | 27 |  | 13 | | 38 | | 12 | 43 | 10 | 52 |
| B: Biofilm; C: Control; **: The Highest RE/Increase %, *: The Lowest RE/Increase %, D^a^: Decrease % | | | | | | | | | | | | | | | | |

Table S3 Residual Levels and Increase/ Removal Efficiency% (I/ RE) of the Tested Parameters in the Seawater after Continuous Treatment Using Unmodified Cellulose Membrane Sheets Filter System.

|  |  | | Flow Rate (mL/h) | | | | | | | | | | | | | | | | | | | | | | | |
| --- | --- | --- | --- | --- | --- | --- | --- | --- | --- | --- | --- | --- | --- | --- | --- | --- | --- | --- | --- | --- | --- | --- | --- | --- | --- | --- |
|  | 200 | | 400 | | 600 | | 200 | | 400 | | 600 |  | | 200 | | | | | 400 | | | | | 600 | | |
|  |  |  |  |  |  |  |  |  |  |  |  |  |  | RC | | I% | | | RC | | I% | | | RC | | I% |
| 0 | Temp.: 26.5 ºC | | | | | | pH: 8.5 | | | | |  |  | DO: 2.0 (mg/L) | | | | | | | | | | | | |
| 1 | 26.4 | | 25 | | 24 | | 8.4 | | 8.3 | | 8.2 |  |  | 2.4 | | 20* | | | 2.7 | | 35* | | | 3 | | 50* |
| 2 | 25.0 | | 25 | | 24 | | 8.4 | | 8.3 | | 8.2 |  |  | 2.7 | | 35 | | | 3.3 | | 65 | | | 3.4 | | 70 |
| 3 | 24.5 | | 25 | | 24 | | 8.3 | | 8.2 | | 8.1 |  |  | 3.1 | | 55 | | | 3.7 | | 85 | | | 3.8 | | 90 |
| 4 | 25.0 | | 25 | | 24 | | 8.3 | | 8.2 | | 8.1 |  |  | 3.5 | | 75** | | | 4.1 | | 105** | | | 4.3 | | 115** |
|  | | | | | | | | | | | | | | | | | | | | | | | | | | |
|  | Raw TDS: 34000 (mg/L) | | | | | | | | | | |  | Raw EC: 68 (ms/cm) | | | | | | | | | | | | | |
|  | 200 | | | 400 | | | | 600 | | | |  | 200 | | | | 400 | | | | | 600 | | | | |
|  | RC | I % | | RC | | I% | | RC | | I% | |  | RC | | I% | | | RC | | I% | | | RC | | I% | |
| 1 | 33000 | 2.9* | | 33000 | | 2.9 | | 33000 | | 2.9 | |  | 66 | | 2.9* | | | 66 | | 2.9 | | | 66 | | 2.9 | |
| 2 | 34000 | - | | 34000 | | - | | 33000 | | 2.9 | |  | 68 | | - | | | 68 | | - | | | 66 | | 2.9 | |
| 3 | 32000 | 5.8** | | 33000 | | 2.9 | | 34000 | | - | |  | 64 | | 5.8** | | | 66 | | 2.9 | | | 68 | | - | |
| 4 | 33000 | 2.9 | | 33000 | | 2.9 | | 34000 | | - | |  | 66 | | 2.9 | | | 66 | | 2.9 | | | 68 | | - | |
|  | | | | | | | | | | | | | | | | | | | | | | | | | | |
|  | Raw Salinity: 45946 (mg/L) | | | | | | | | | | |  | | Raw TSS: 1100 (mg/L) | | | | | | | | | | | | |
|  | 200 | | | | 400 | | | | 600 | | |  |  | 200 | | | | | 400 | | | | | 600 | | |
|  | RC | | I% | | RC | | I% | | RC | | I% |  |  | RC | | RE% | | | RC | | RE% | | | RC | | RE% |
| 1 | 44478 | | 3.1* | | 44478 | | 3.1 | | 44478 | | 3.1 |  |  | 1000 | | 9* | | | 850 | | 23* | | | 730 | | 34* |
| 2 | 45946 | | - | | 45946 | | - | | 44478 | | 3.1 |  |  | 870 | | 21 | | | 720 | | 35 | | | 610 | | 45 |
| 3 | 43014 | | 6.3** | | 44478 | | 3.1 | | 45946 | | - |  |  | 800 | | 27 | | | 600 | | 45 | | | 560 | | 49 |
| 4 | 44478 | | 3.1 | | 44478 | | 3.1 | | 45946 | | - |  |  | 700 | | 36** | | | 500 | | 55** | | | 440 | | 60** |
|  | | | | | | | | | | | | | | | | | | | | | | | | | | |
|  | Raw COD: 33 (mg/L) | | | | | | | | | | |  | | Raw BOD: 22 (mg/L) | | | | | | | | | | | | |
|  | 200 | | | | 400 | | | | 600 | | |  |  | 200 | | | | | 400 | | | | | 600 | | |
|  | RC | | RE% | | RC | | RE% | | RC | | RE% |  |  | RC | | RE% | | | RC | | RE% | | | RC | | RE% |
| 1 | 32 | | 3* | | 30 | | 9* | | 27 | | 18* |  |  | 21 | | 5* | | | 19 | | 14* | | | 18 | | 18* |
| 2 | 29 | | 12 | | 27 | | 18 | | 24 | | 27 |  |  | 19 | | 14 | | | 17 | | 23 | | | 16 | | 27 |
| 3 | 27 | | 18 | | 24 | | 27 | | 22 | | 33 |  |  | 17 | | 23 | | | 15 | | 32 | | | 14 | | 36 |
| 4 | 25 | | 24** | | 23 | | 30** | | 21 | | 36** |  |  | 16 | | 27** | | | 14 | | 36** | | | 12 | | 45** |
| ** The Highest RE/I %, * The Highest RE/I % | | | | | | | | | | | | | | | | | | | | | | | | | | |

Table S4 Residual Levels and Increase/ Removal Efficiency% (I/ RE) of the Tested Parameters in the Seawater after Continuous Treatment Using AgNPs/AC-NC / Gravel Biofilm System.

|  |  | | Flow Rate (mL/h) | | | | | | | | | | | | | | | | | | | | | | | |
| --- | --- | --- | --- | --- | --- | --- | --- | --- | --- | --- | --- | --- | --- | --- | --- | --- | --- | --- | --- | --- | --- | --- | --- | --- | --- | --- |
|  | 200 | | 400 | | 600 | | 200 | | 400 | | 600 |  | | 200 | | | | | 400 | | | | | 600 | | |
|  |  |  |  |  |  |  |  |  |  |  |  |  |  | RC | | I% | | | RC | | I% | | | RC | | I% |
| 0 | Temp.: 26 ºC | | | | | | pH: 8.4 | | | | |  |  | DO: 2.5 (mg/L) | | | | | | | | | | | | |
| 1 | 26.3 | | 26.8 | | 27.0 | | 8.3 | | 8.2 | | 8.3 |  |  | 2.7 | | 8* | | | 2.9 | | 16* | | | 3.2 | | 28* |
| 2 | 26.7 | | 26.8 | | 27.0 | | 8.3 | | 8.3 | | 8.2 |  |  | 3.2 | | 28 | | | 3.5 | | 40 | | | 3.7 | | 48 |
| 3 | 26.7 | | 27.0 | | 27.0 | | 8.3 | | 8.3 | | 8.3 |  |  | 3.7 | | 48 | | | 3.9 | | 56 | | | 4.2 | | 68 |
| 4 | 26.9 | | 27.0 | | 27.0 | | 8.3 | | 8.2 | | 8.2 |  |  | 4.0 | | 60** | | | 4.3 | | 72** | | | 4.7 | | 88** |
|  | | | | | | | | | | | | | | | | | | | | | | | | | | |
|  | Raw TDS: 36000 (mg/L) | | | | | | | | | | |  | Raw EC: 72 (ms/cm) | | | | | | | | | | | | | |
|  | 200 | | | 400 | | | | 600 | | | |  | 200 | | | | 400 | | | | | 600 | | | | |
|  | RC | I % | | RC | | I% | | RC | | I% | |  | RC | | I% | | | RC | | I% | | | RC | | I% | |
| 1 | 37000 | 2.7 | | 36000 | | - | | 37000 | | 2.7 | |  | 74 | | 2.7 | | | 72 | | - | | | 74 | | 2.7 | |
| 2 | 36000 | - | | 36000 | | - | | 36000 | | - | |  | 72 | | - | | | 72 | | - | | | 72 | | - | |
| 3 | 35000 | -2.7 | | 36000 | | - | | 36000 | | - | |  | 70 | | -2.7 | | | 72 | | - | | | 72 | | - | |
| 4 | 36000 | - | | 36000 | | - | | 37000 | | 2.7 | |  | 72 | | - | | | 72 | | - | | | 74 | | 2.7 | |
|  | | | | | | | | | | | | | | | | | | | | | | | | | | |
|  | Raw Salinity: 48789 (mg/L) | | | | | | | | | | |  | | Raw TSS: 1200 (mg/L) | | | | | | | | | | | | |
|  | 200 | | | | 400 | | | | 600 | | |  |  | 200 | | | | | 400 | | | | | 600 | | |
|  | RC | | I% | | RC | | I% | | RC | | I% |  |  | RC | | RE% | | | RC | | RE% | | | RC | | RE% |
| 1 | 50265 | | 3.0** | | 48789 | | - | | 50265 | | 3.0** |  |  | 890 | | 26* | | | 950 | | 21* | | | 1050 | | 13* |
| 2 | 48789 | | - | | 48789 | | - | | 48789 | | - |  |  | 850 | | 29 | | | 880 | | 27 | | | 930 | | 23 |
| 3 | 47317 | | -3.0 | | 48789 | | - | | 48789 | | - |  |  | 780 | | 35 | | | 810 | | 33 | | | 850 | | 29 |
| 4 | 48789 | | - | | 48789 | | - | | 50265 | | 3.0 |  |  | 700 | | 42** | | | 745 | | 38** | | | 820 | | 32** |
|  | | | | | | | | | | | | | | | | | | | | | | | | | | |
|  | Raw COD: 30 (mg/L) | | | | | | | | | | |  | | Raw BOD: 20 (mg/L) | | | | | | | | | | | | |
|  | 200 | | | | 400 | | | | 600 | | |  |  | 200 | | | | | 400 | | | | | 600 | | |
|  | RC | | RE% | | RC | | RE% | | RC | | RE% |  |  | RC | | RE% | | | RC | | RE% | | | RC | | RE% |
| 1 | 28 | | 7* | | 27 | | 10* | | 26 | | 13* |  |  | 19 | | 5* | | | 17 | | 15* | | | 14 | | 30* |
| 2 | 26 | | 13 | | 25 | | 23 | | 24 | | 20 |  |  | 17 | | 15 | | | 15 | | 25 | | | 12 | | 40 |
| 3 | 23 | | 23 | | 22 | | 27 | | 20 | | 33 |  |  | 16 | | 20 | | | 13 | | 35 | | | 11 | | 45 |
| 4 | 21 | | 30** | | 20 | | 33** | | 19 | | 37** |  |  | 15 | | 25** | | | 11 | | 45** | | | 10 | | 50** |
| ** The Highest RE/I %, * The Highest RE/I % | | | | | | | | | | | | | | | | | | | | | | | | | | |

Table S5 Residual Levels and Increase/ Removal Efficiency% (I/ RE) of the Tested Parameters in the Seawater after Continuous Treatment Using AgNPs/AC-NC Modified Cellulose Membrane/ Gravel Biofilm Filter System.

| Time  (h) | Temperature ºC | | | pH | | |  | DO | | | | | |
| --- | --- | --- | --- | --- | --- | --- | --- | --- | --- | --- | --- | --- | --- |
|  | Flow Rate (mL/h) | | | | | | | | | | | | |
|  | 200 | 400 | 600 | 200 | 400 | 600 |  | 200 | | 400 | | 600 | |
|  |  |  |  |  |  |  |  | RC | I% ^a^ | RC | I% | RC | I% |
| 0 | Temp.: 26 ºC | | | pH: 8.6 | | |  | DO: 2.4 (mg/L) | | | | | |
| 1 | 26.5 | 26.5 | 27 | 8.5 | 8.2 | 8.3 |  | 2.8 | 17* | 2.9 | 21* | 3.1 | 29* |
| 2 | 27 | 26.8 | 27 | 8.4 | 8.3 | 8.4 |  | 3.3 | 38 | 3.4 | 42 | 3.6 | 50 |
| 3 | 27 | 26.5 | 27 | 8.3 | 8.4 | 8.3 |  | 3.7 | 54 | 3.9 | 63 | 4.0 | 67 |
| 4 | 27 | 26.9 | 27 | 8.3 | 8.4 | 8.4 |  | 4.2 | 75** | 4.4 | 83** | 4.6 | 92** |
|  |  | | | | | | | | | | | | |
|  | Raw TDS: 36000 (mg/L) | | | | | |  | Raw EC: 72 (ms/cm) | | | | | |
|  | 200 | | 400 | | 600 | |  | 200 | | 400 | | 600 | |
|  | RC | I% | RC | I% | RC | I% |  | RC | I% ^a^ | RC | I% ^a^ | RC | I% ^a^ |
| 1 | 37000 | 2.7** | 36000 | - | 36000 | - |  | 74 | 2.7** | 72 | - | 72 | - |
| 2 | 36000 | - | 36000 | - | 36000 | - |  | 72 | - | 72 | - | 72 | - |
| 3 | 36000 | - | 36000 | - | 36000 | - |  | 72 | - | 72 | - | 72 | - |
| 4 | 37000 | 2.7** | 36000 | - | 36000 | - |  | 74 | 2.7** | 72 | - | 72 | - |
|  | | | | | | | | | | | | | |
|  | Raw Salinity: 48789 (mg/L) | | | | | |  | Raw TSS: 1300 (mg/L) | | | | | |
|  | 200 | | 400 | | 600 | |  | 200 | | 400 | | 600 | |
|  | RC | I% | RC | I% | RC | I% |  | RC | RE% | RC | RE% | RC | RE% |
| 1 | 50265 | 3 | 48789 | - | 48789 | - |  | 1000 | 23* | 1100 | 15* | 1200 | 8* |
| 2 | 48789 | - | 48789 | - | 48789 | - |  | 890 | 32 | 920 | 29 | 970 | 25 |
| 3 | 48789 | - | 48789 | - | 48789 | - |  | 840 | 35 | 880 | 32 | 910 | 30 |
| 4 | 50265 | 3 | 48789 | - | 48789 | - |  | 750 | 42** | 790 | 39** | 850 | 35** |
|  | | | | | | | | | | | | | |
|  | Raw COD: 28 (mg/L) | | | | | |  | Raw BOD: 18 (mg/L) | | | | | |
|  | 200 | | 400 | | 600 | |  | 200 | | 400 | | 600 | |
|  | RC | RE | RC | RE | RC | RE% |  | RC | RE% | RC | RE% | RC | RE% |
| 1 | 26 | 7* | 24 | 14* | 18 | 36* |  | 17 | 6* | 14 | 22* | 11 | 39* |
| 2 | 24 | 14 | 21 | 25 | 17 | 39 |  | 16 | 11 | 12 | 33 | 9 | 50 |
| 3 | 20 | 29 | 16 | 43 | 15 | 46 |  | 14 | 22 | 11 | 39 | 8 | 56 |
| 4 | 19 | 32** | 15 | 46** | 14 | 50** |  | 12 | 33** | 10 | 44** | 6 | 67** |
| ** The Highest RE/Increase %, * The Lowest RE/Increase % | | | | | | | | | | | | | |

Table 6S Residual Levels and Increase/ Removal Efficiency% (I/ RE) of the Tested Parameters

in the Sea Water after Continuous Treatment Using AgNPs/AC-NC Modified

Cellulose Membrane/ Gravel Filter System.

| Time  (h) | | Temperature ºC | | | pH | | |  | DO | | | | | |
| --- | --- | --- | --- | --- | --- | --- | --- | --- | --- | --- | --- | --- | --- | --- |
|  |  | Flow Rate (mL/h) | | | | | | | | | | | | |
|  |  | 200 | 400 | 600 | 200 | 400 | 600 |  | 200 | | 400 | | 600 | |
|  |  |  |  |  |  |  |  |  | RC | I% ^a^ | RC | I% | RC | I% |
| 0 | | Temp.: 26 ºC | | | pH: 8.1 | | |  | DO: 3.0 (mg/L) | | | | | |
| 1 | | 26.5 | 27 | 28 | 8.3 | 8.2 | 8.4 |  | 3.3 | 10 | 4.2 | 40 | 5.6 | 87 |
| 2 | | 27 | 27 | 28 | 8.4 | 8.3 | 8.3 |  | 3.5 | 17 | 4.5 | 50 | 5.8 | 93 |
| 3 | | 27 | 27 | 28 | 8.3 | 8.4 | 8.4 |  | 3.7 | 23 | 4.8 | 60 | 6.3 | 110 |
| 4 | | 27 | 27 | 28 | 8.2 | 8.4 | 8.3 |  | 4.0 | 33^a^ | 5.3 | 77 ^a^ | 6.6 | 120 ^a^ |
|  |  | | | | | | | | | | | | | |
|  | | Raw TDS: 36000 (mg/L) | | | | | |  | Raw EC: 72 (ms/cm) | | | | | |
|  |  | 200 | | 400 | | 600 | |  | 200 | | 400 | | 600 | |
|  |  | RC | I% | RC | I% | RC | I% |  | RC | I% ^a^ | RC | I% ^a^ | RC | I% ^a^ |
| 1 | | 37000 | 2.7 | 37000 | 2.7 ^a^ | 36000 | 0.0 |  | 74 | 2.7 | 74 | 2.7 ^a^ | 72 | 0.0 |
| 2 | | 38000 | 5.5 ^a^ | 37000 | 2.7 | 36000 | 0.0 |  | 76 | 5.5 ^a^ | 74 | 2.7 | 72 | 0.0 |
| 3 | | 36000 | 0.0 | 37000 | 2.7 | 37000 | 2.7 ^a^ |  | 72 | 0.0 | 74 | 2.7 | 74 | 2.7 ^a^ |
| 4 | | 37000 | 2.7 | 37000 | 2.7 | 37000 | 2.7 |  | 74 | 2.7 | 74 | 2.7 | 74 | 2.7 |
|  | | | | | | | | | | | | | | |
|  | | Raw Salinity: 48789 (mg/L) | | | | | |  | Raw TSS: 1060 (mg/L) | | | | | |
|  |  | 200 | | 400 | | 600 | |  | 200 | | 400 | | 600 | |
|  |  | RC | I% | RC | I% | RC | I% |  | RC | RE% | RC | RE% | RC | RE% |
| 1 | | 50265 | 3 | 50265 | 3 ^a^ | 48789 | 0.0 |  | 1010 | 4.7 | 865 | 18 | 750 | 29 |
| 2 | | 51856 | 6 ^a^ | 50265 | 3 | 48789 | 0.0 |  | 970 | 8.4 | 830 | 22 | 730 | 31 |
| 3 | | 48789 | 0. 00.0 | 50265 | 3 | 50265 | 3 ^a^ |  | 930 | 12.0 | 810 | 24 | 710 | 33 |
| 4 | | 50265 | 3 | 50265 | 3 | 50265 | 3 |  | 900 | 15^b^ | 785 | 26 ^b^ | 690 | 35 ^b^ |
|  | | | | | | | | | | | | | | |
|  | | Raw COD: 31 (mg/L) | | | | | |  | Raw BOD: 19 (mg/L) | | | | | |
|  | | 200 | | 400 | | 600 | |  | 200 | | 400 | | 600 | |
|  | | RC | RE% | RC | RE% | RC | RE% |  | RC | RE% | RC | RE% | RC | RE% |
| 1 | | 30 | 3.2 | 24 | 23 | 20 | 35 |  | 18 | 5.2 | 14 | 26 | 10 | 47 |
| 2 | | 29 | 6.4 | 23 | 26 | 19 | 39 |  | 17 | 11 | 13 | 32 | 9 | 53 |
| 3 | | 27 | 13 | 22 | 29 | 18 | 42 |  | 16 | 16 | 12 | 37 | 8 | 58 |
| 4 | | 25 | 19 ^b^ | 21 | 32 ^b^ | 17 | 45 ^b^ |  | 15 | 21 ^b^ | 11 | 42 ^b^ | 7 | 63 ^b^ |
| ^a^ The Highest Increase %, ^b^ The Highest RE % | | | | | | | | | | | | | | |
