# Supplementary material for: Desalination of seawater using integrated microbial biofilm/cellulose acetate membrane and silver NPs/activated carbon nanocomposite in a continuous mode
Source: Sci Rep. 2024 Jan 2;14:274. doi: 10.1038/s41598-023-50311-0 (PMC10762133; doi:10.1038/s41598-023-50311-0)
Supplement: Supplementary file 2 — Supplementary Figures. [file 41598_2023_50311_MOESM2_ESM.pdf]

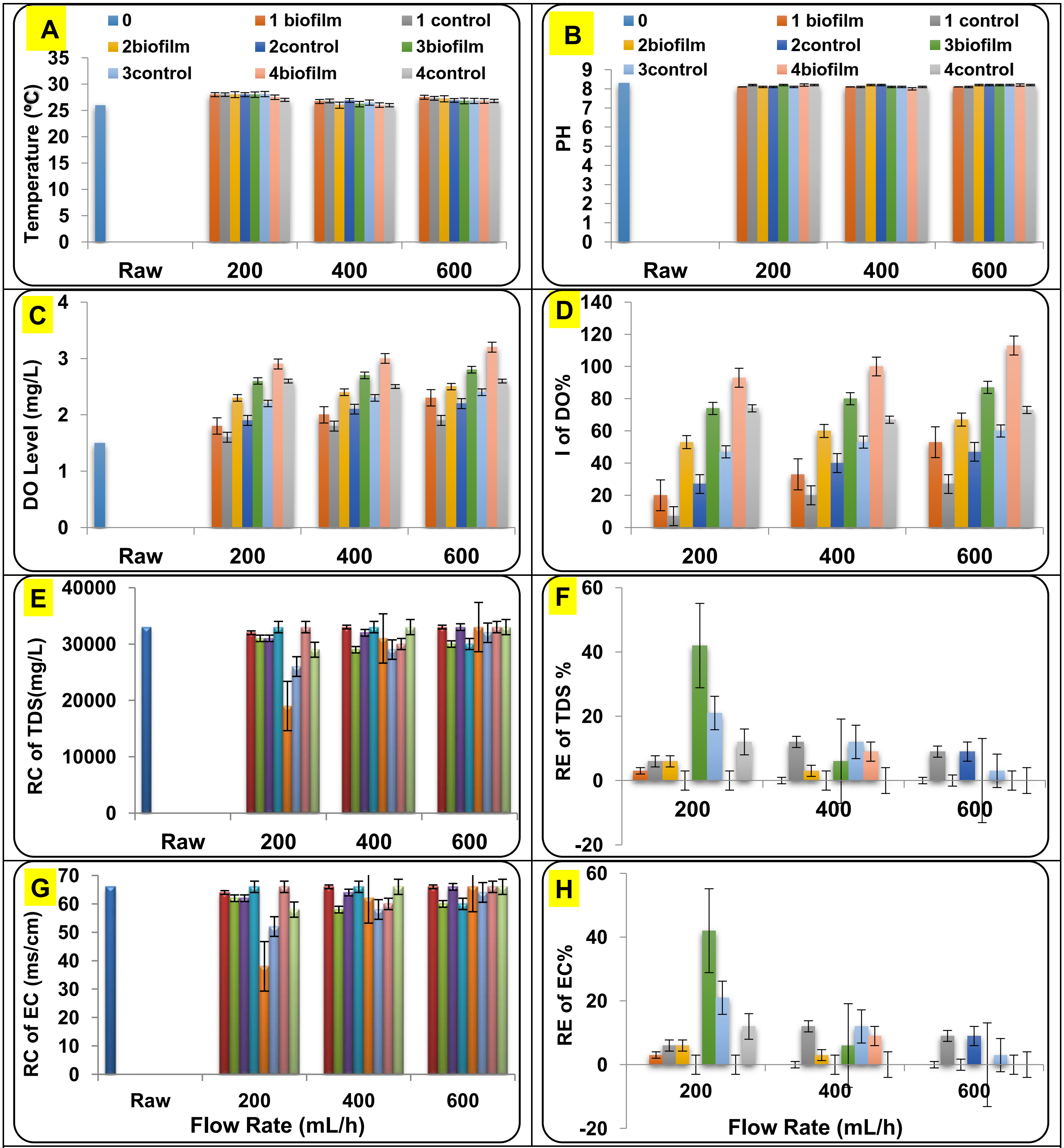

Figure S1. Variations in the Levels and Increase % (I%) of Temperature (A), pH (B), DO (C & D), TDS (E & F) and EC (G & H) after Continuous Treatment Using *Bacillus cereus* Gravel Biofilm System at Different Flow Rates and Running Times.

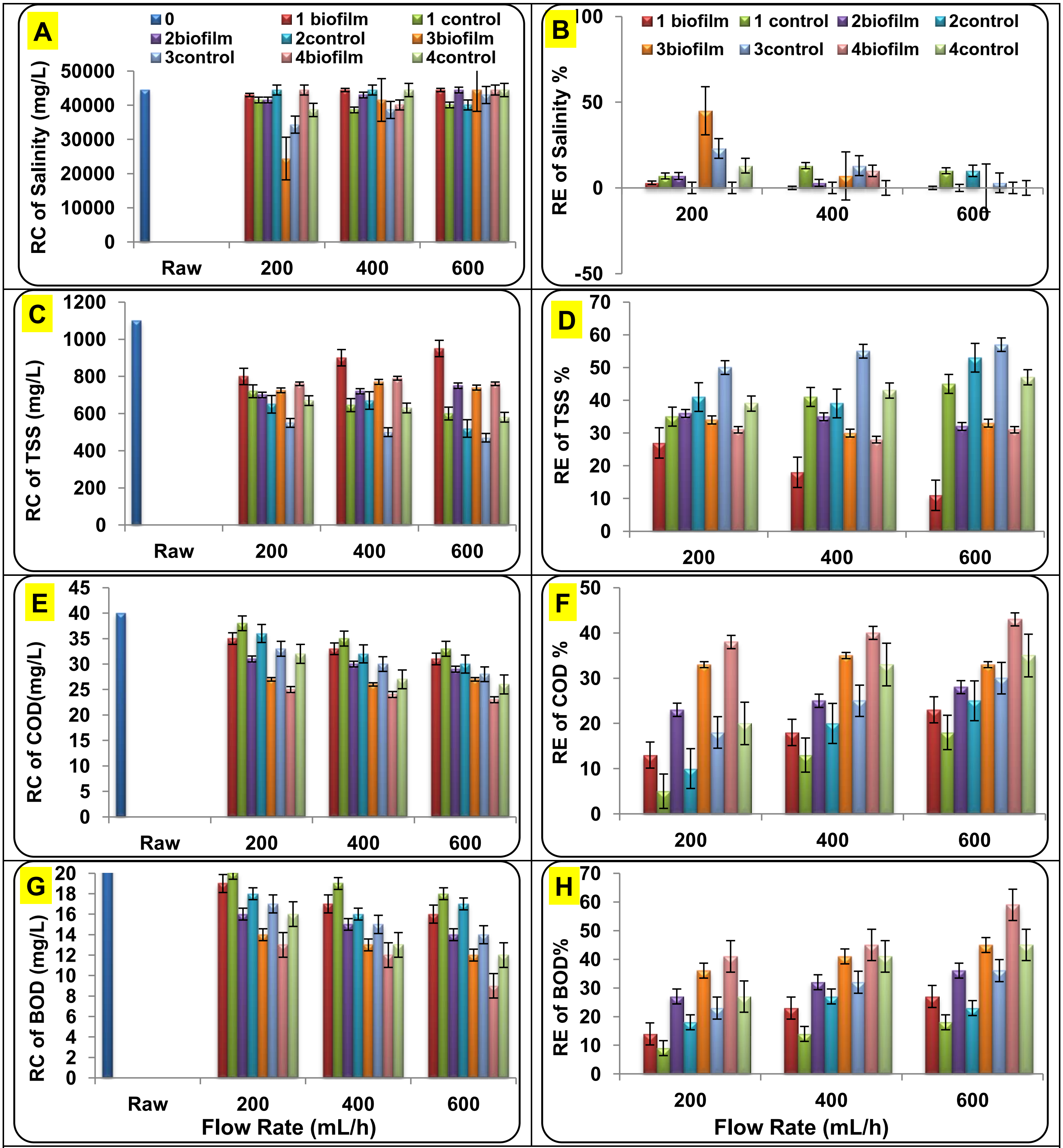

Figure S2. Residual Concentration (RC) and Removal/Increase % (RE/I%) of Salinity (A & B), TSS (C & D), COD (E & F) and BOD (G & H) after Continuous Treatment Using *Bacillus cereus* Gravel Biofilm System at Different Flow Rates and Running Time.

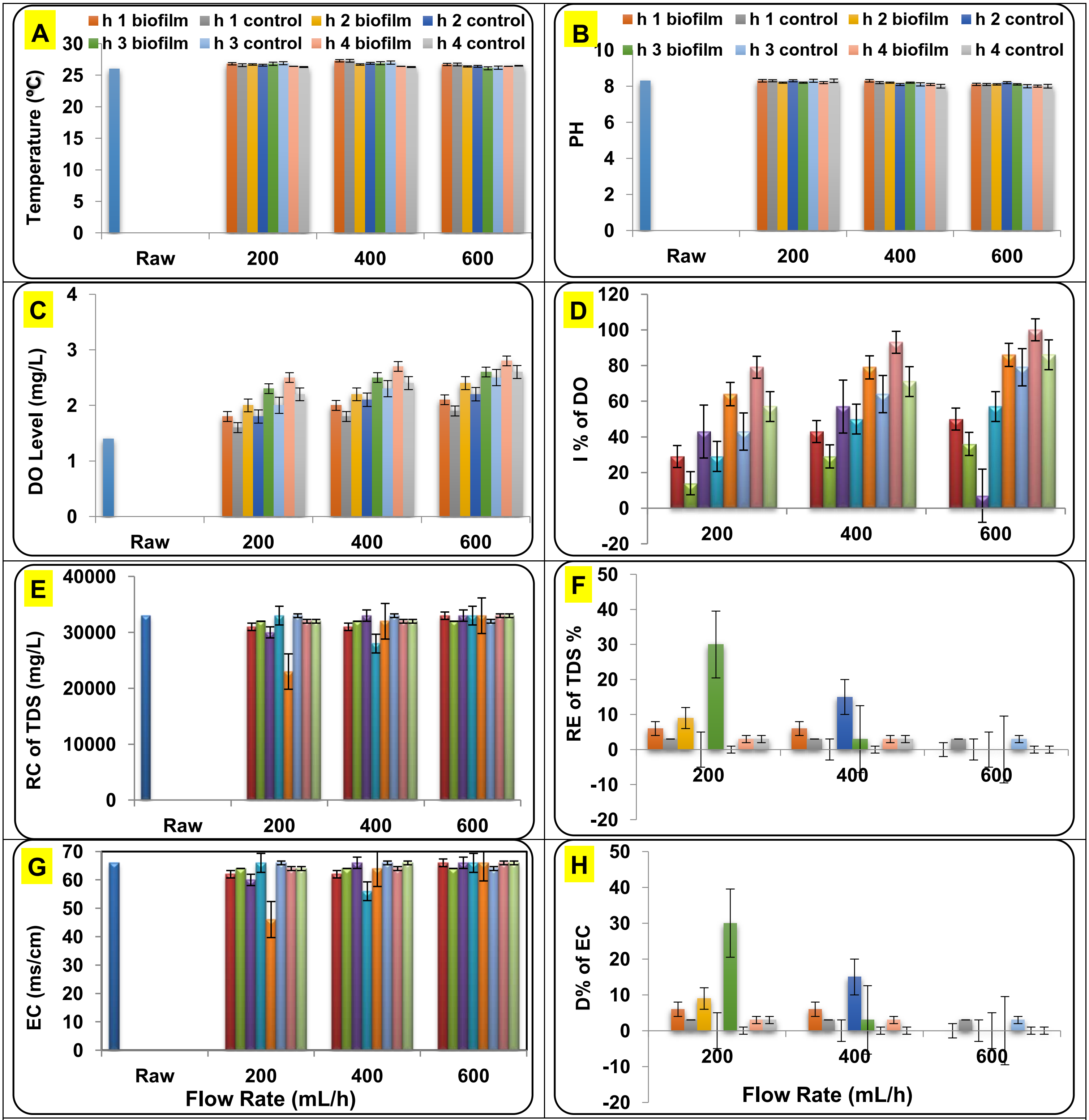

Figure S3. Variations in the Levels and Increase % (I%) of Temperature (A), pH (B), DO (C & D), TDS (E & F) and EC (G & H) after Continuous Treatment Using *Bacillus cereus* Gravel Biofilm and Unmodified Cellulose Membrane Filter System at Different Flow Rates and Running Times.

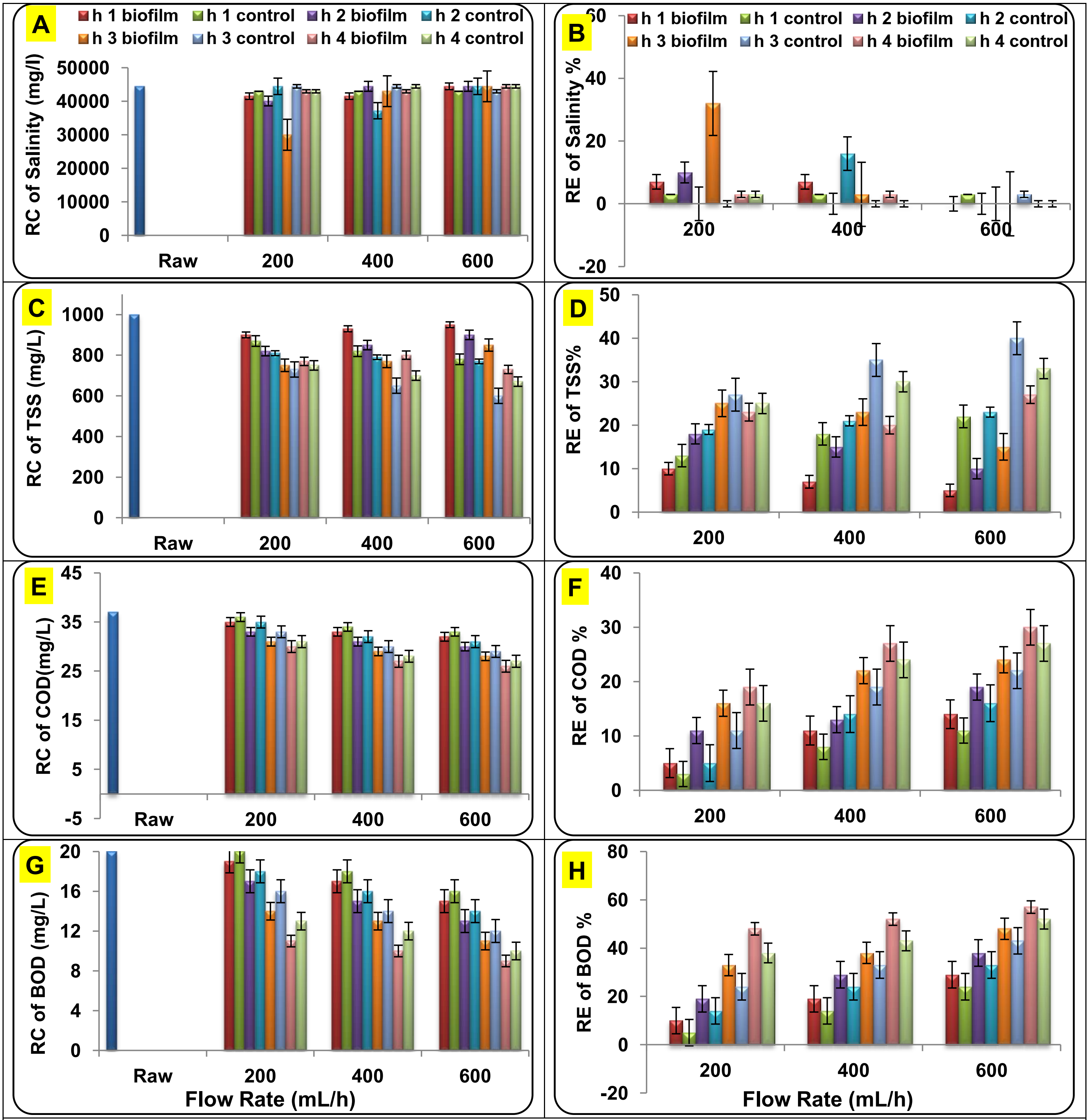

Figure S4. Residual Concentration (RC) and Removal/Increase % (RE/I%) of Salinity (A & B), TSS (C & D), COD (E & F) and BOD (G & H) after Continuous Treatment Using *Bacillus cereus* Gravel Biofilm and Unmodified Cellulose Membrane Filter System at Different Flow Rates and Running Time.

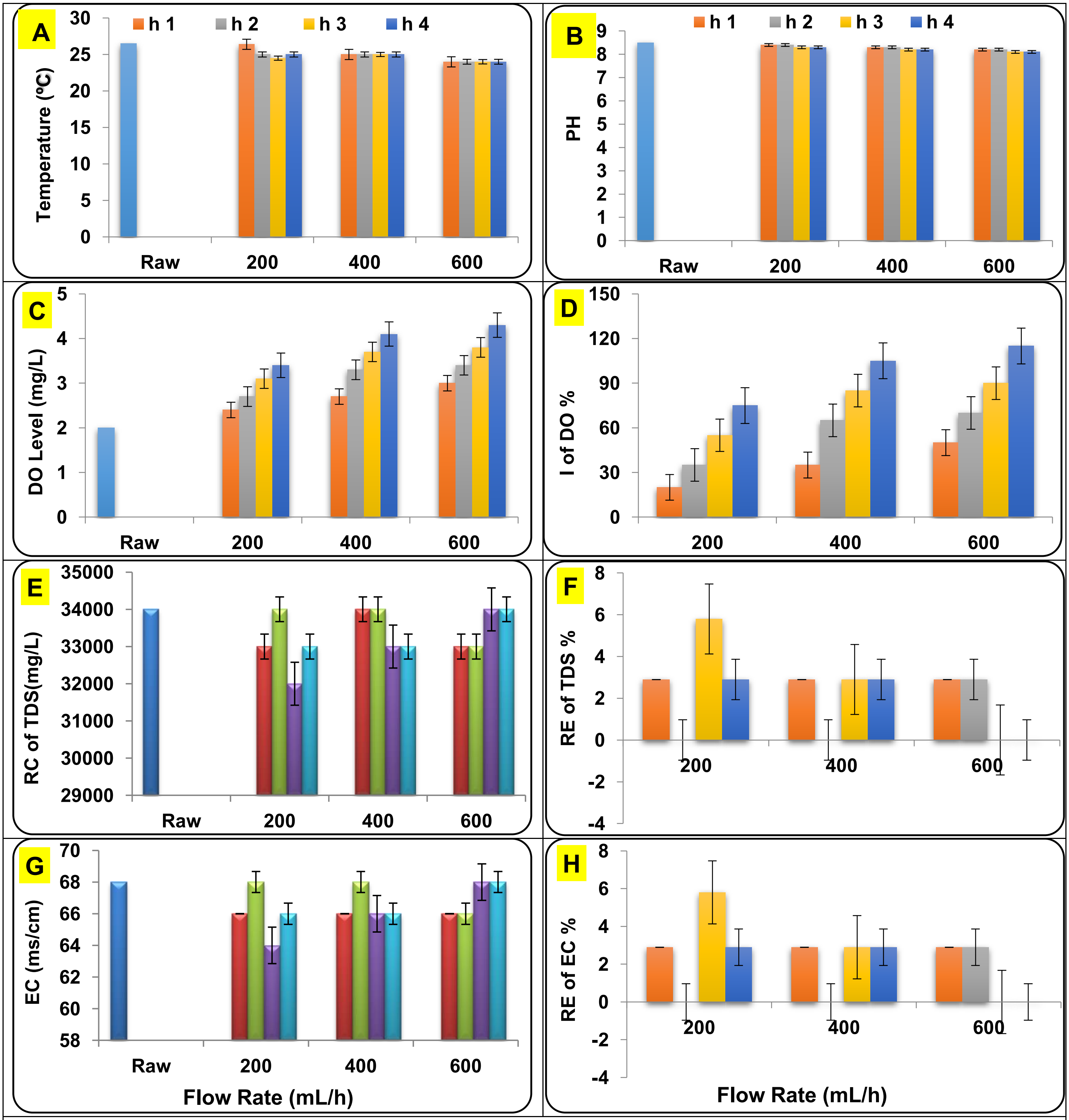

Figure S5. Variations in the Levels and Increase % (I%) of Temperature (A), pH (B), DO (C & D), TDS (E & F) and EC (G & H) after Continuous Treatment Using Unmodified Cellulose Membrane Sheets Filter System at Different Flow Rates and Running Times.

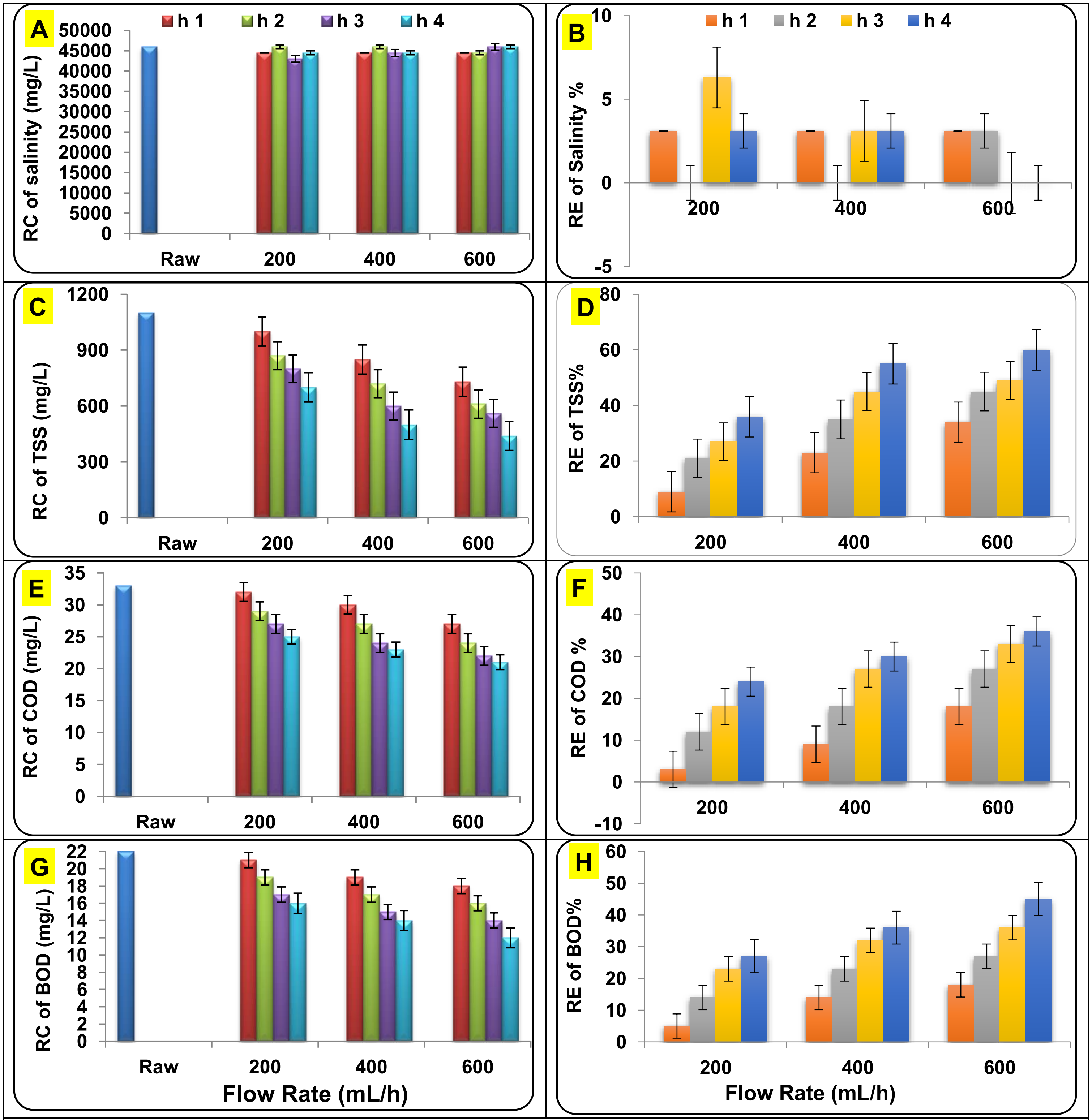

Figure S6. Residual Concentration (RC) and Removal/Increase % (RE/I%) of Salinity (A & B), TSS (C & D), COD (E & F) and BOD (G & H) after Continuous Treatment Using Unmodified Cellulose Membrane Sheets Filter System at Different Flow Rates and Running Time.

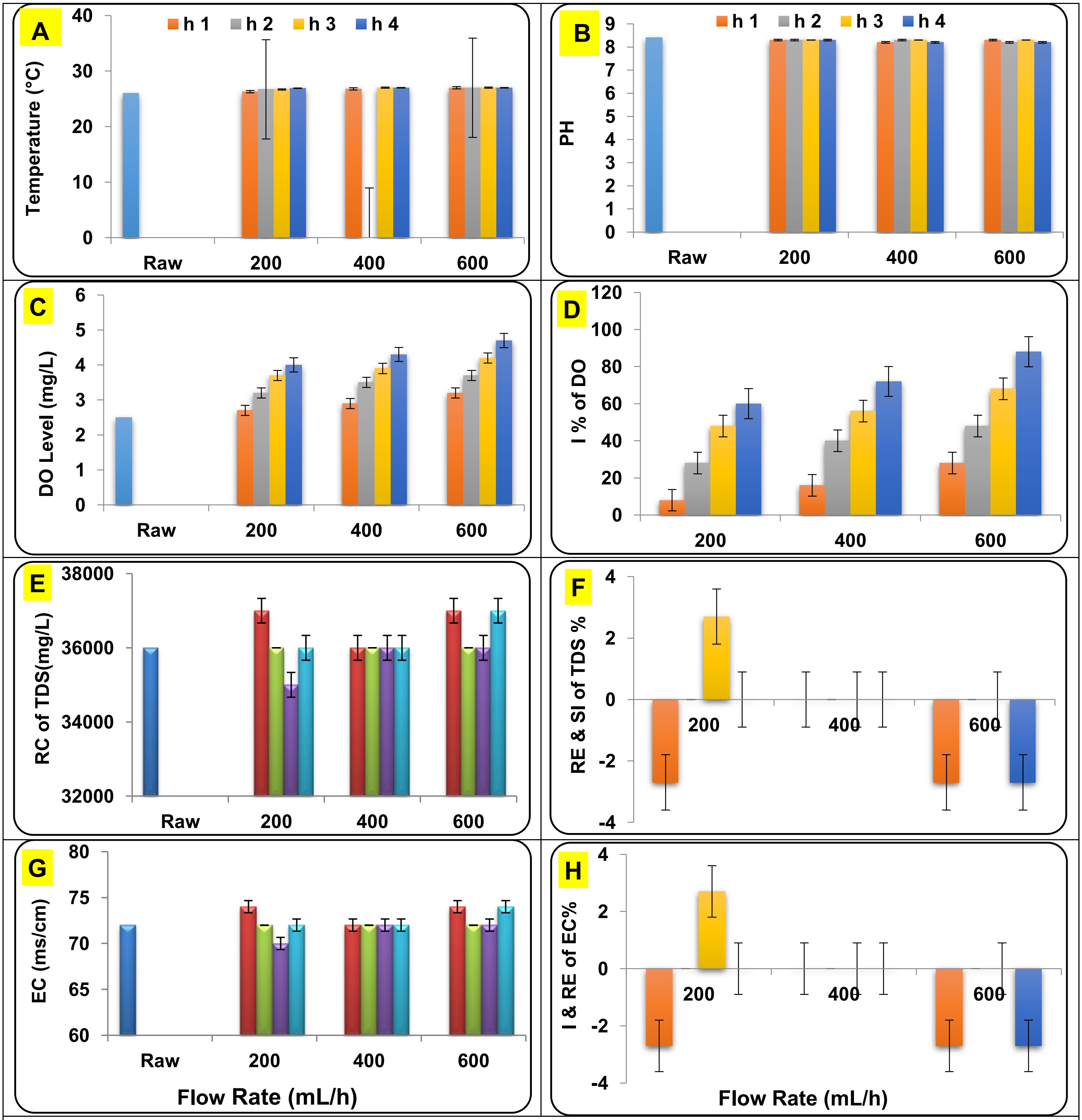

Figure 7S. Variations in the Levels and Increase % (I%) of Temperature (A), pH (B), DO (C & D), TDS (E & F) and EC (G & H) after Continuous Treatment Using AgNPs/AC-NC / Gravel Biofilm System at Different Flow Rates and Running Times.

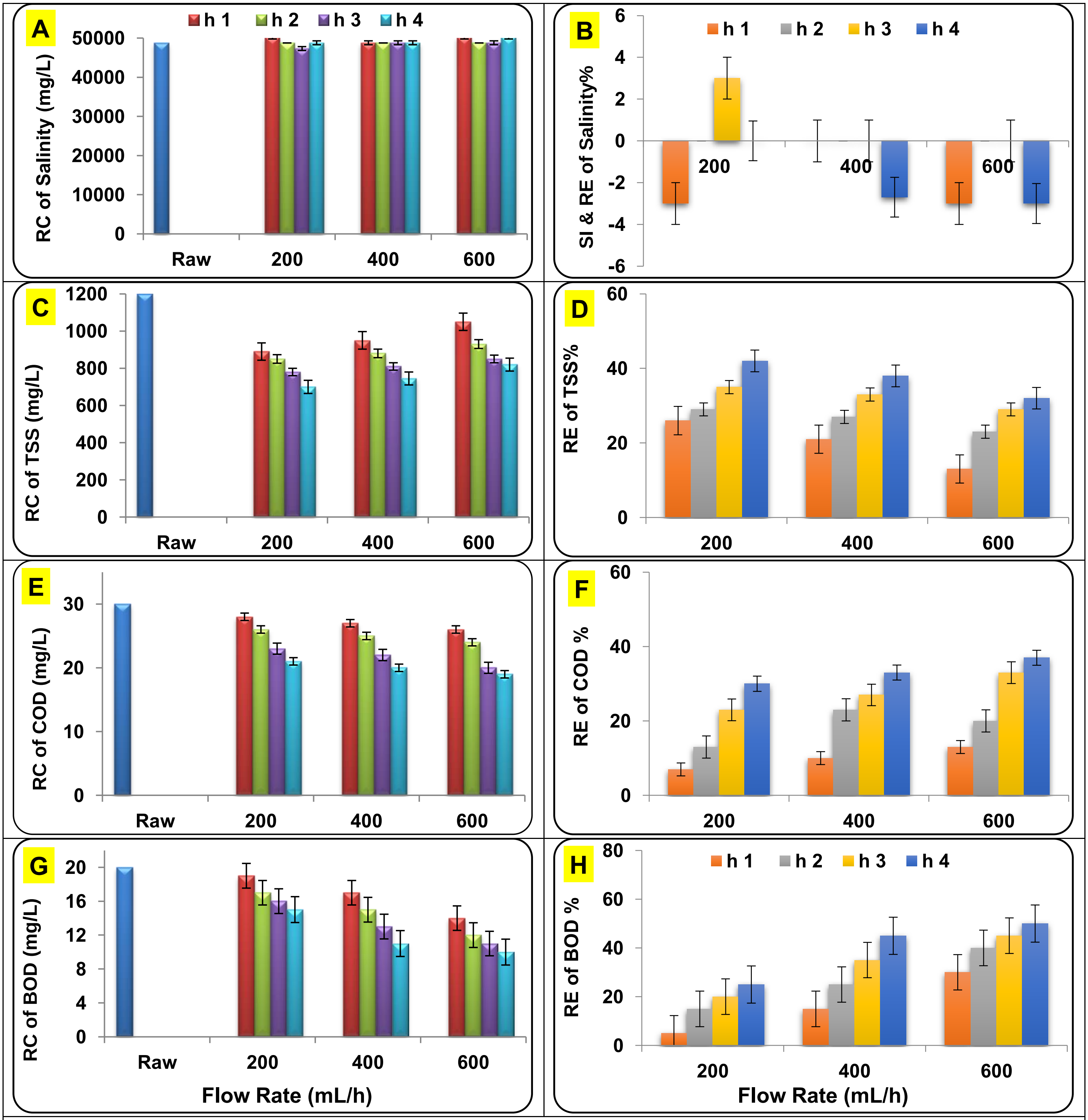

Figure 8S. Residual Concentration (RC) and Removal/Increase % (RE/I%) of Salinity (A & B), TSS (C & D), COD (E & F) and BOD (G & H) after Continuous Treatment Using AgNPs/AC-NC/ Gravel Biofilm System at Different Flow Rates and Running Times.

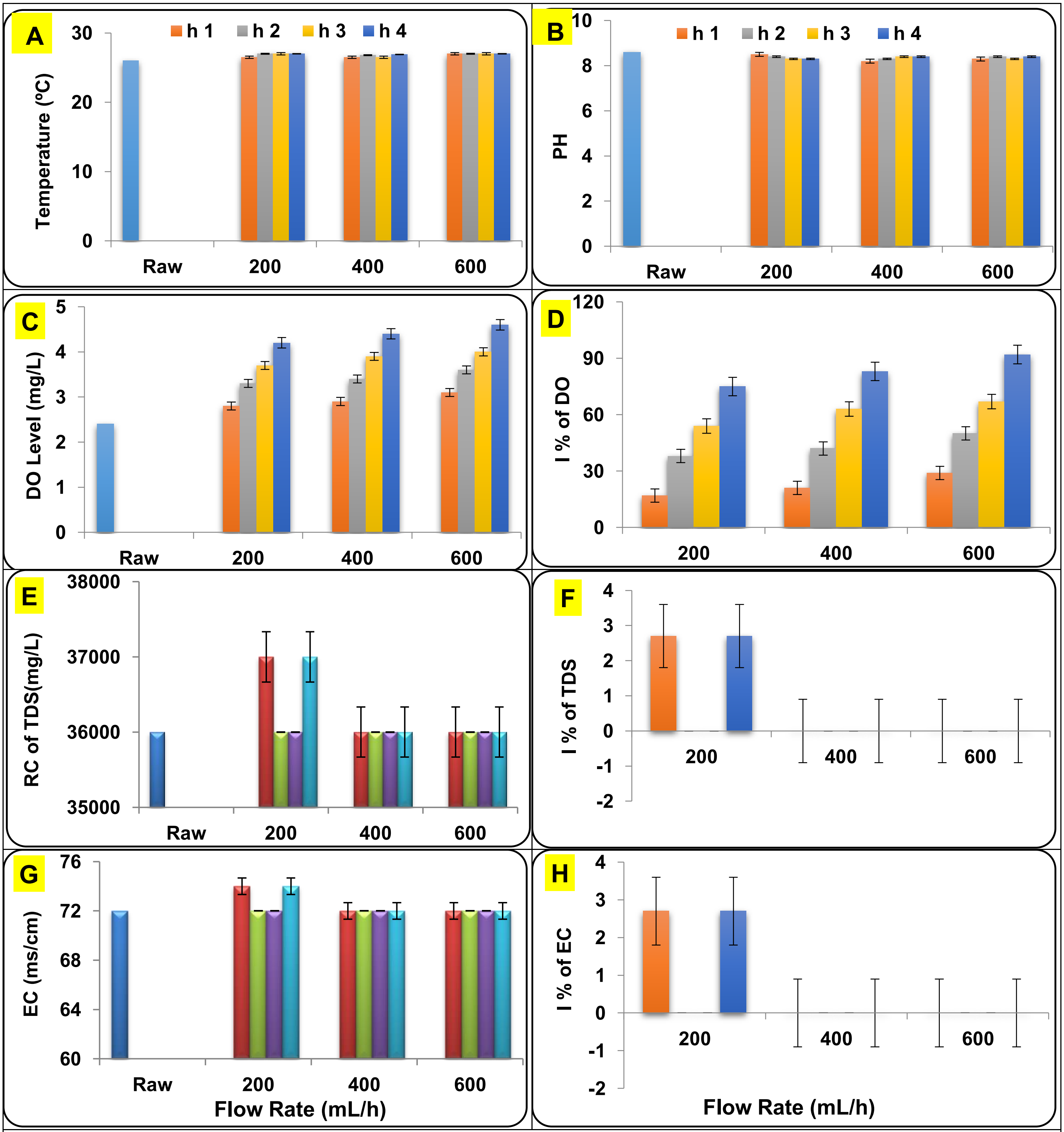

Figure S9. Variations in the Levels and Increase % (I%) of Temperature (A), pH (B), DO (C & D), TDS (E & F) and EC (G & H) after Continuous Treatment Using AgNPs/AC-NC Modified Cellulose Membrane / Gravel Biofilm Filter System at Different Flow Rates and Running Times.

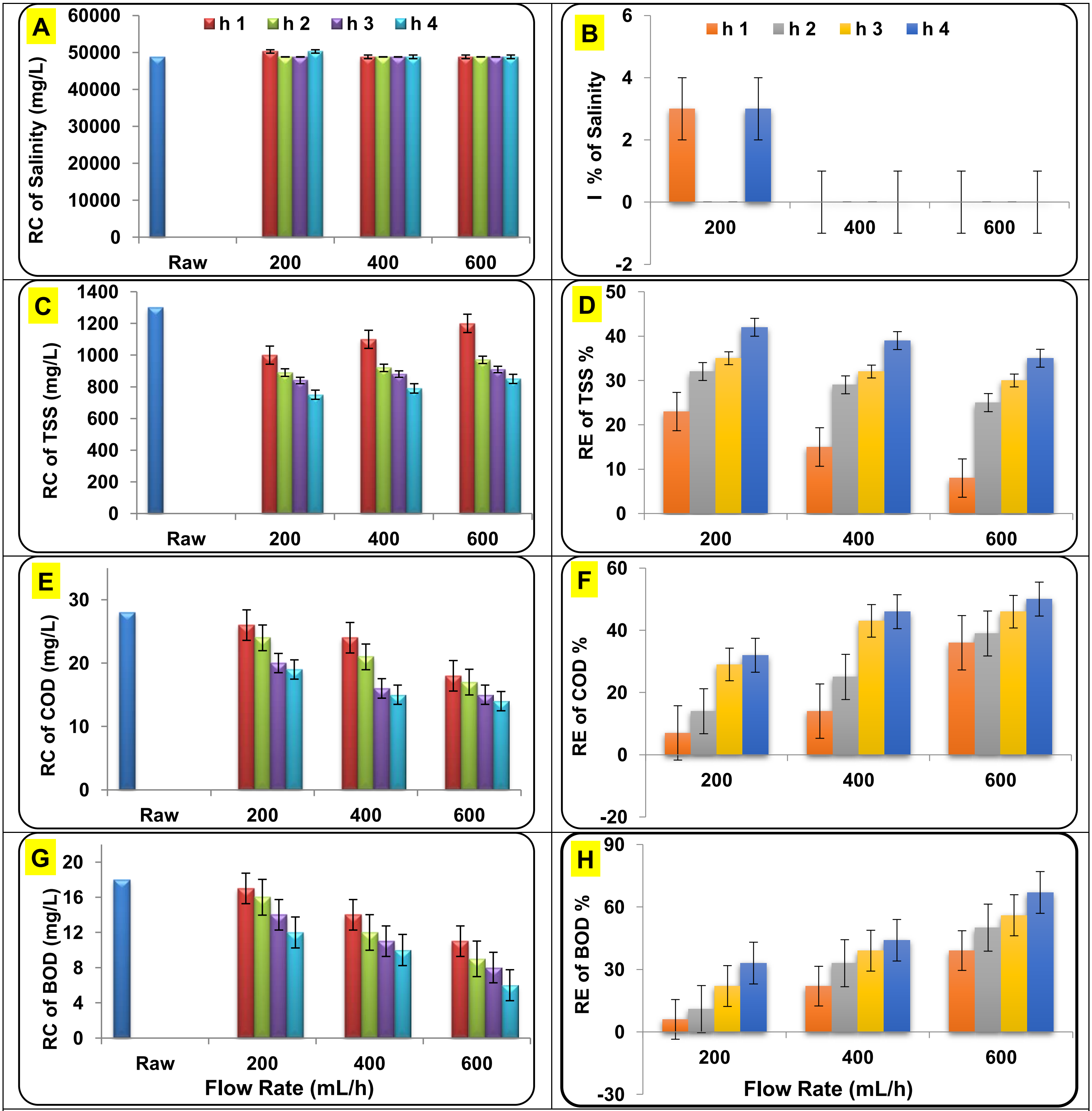

Figure S10. Residual Concentration (RC) and Removal/Increase % (RE/I%) of Salinity (A & B), TSS (C & D), COD (E & F) and BOD (G & H) after Continuous Treatment Using Gravel Biofilm/ AgNPs/AC-NC Modified Cellulose Membrane Filter System at Different Flow Rates and Running Times.

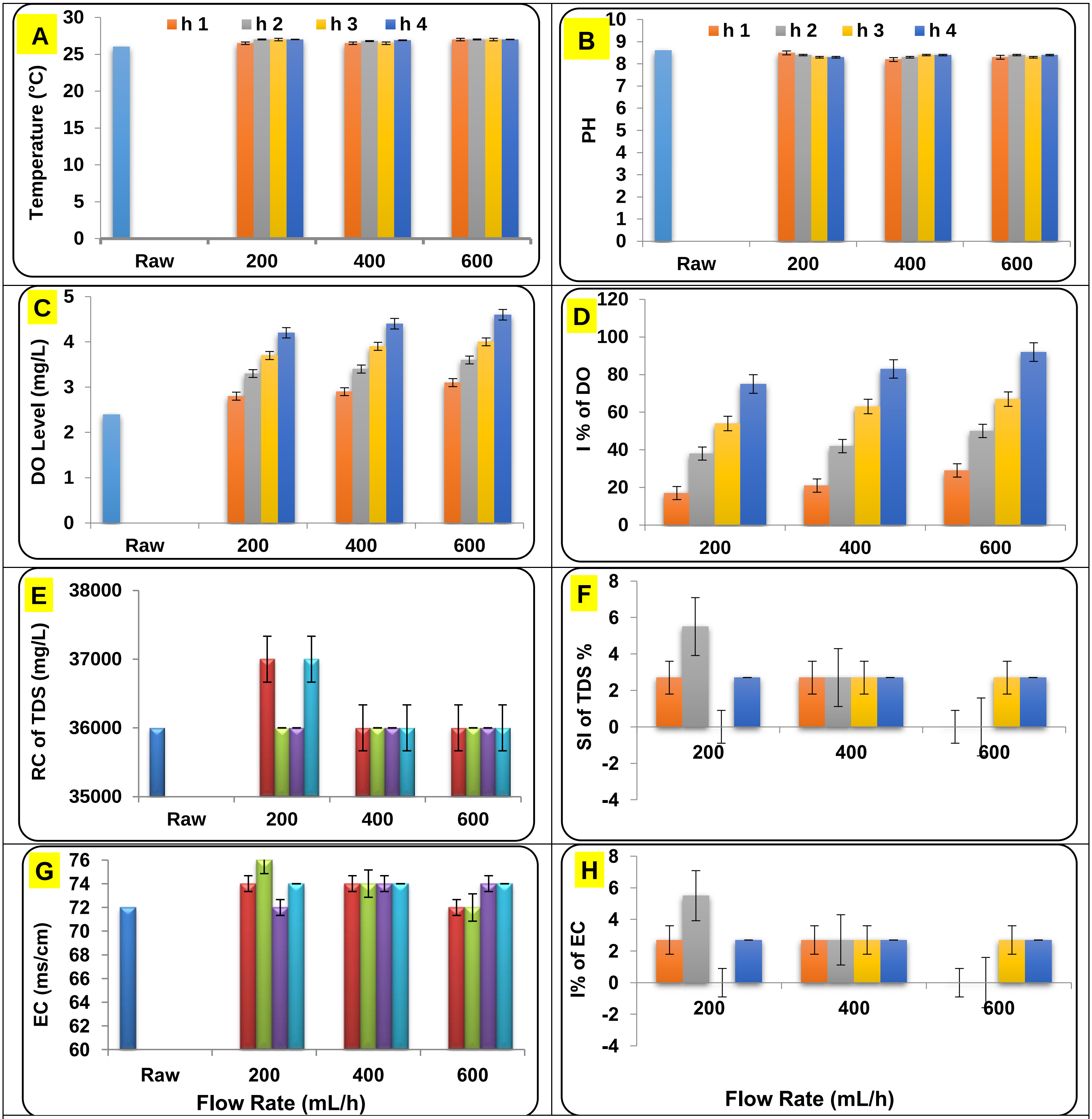

Figure S11. Variations in the Levels and Increase % (I%) of Temperature (A), pH (B), DO (C & D), TDS (E & F) and EC (G & H) after Continuous Treatment Using AgNPs/AC-NC Modified Cellulose Membrane/ Gravel Filter System at Different Flow Rates and Running Times.

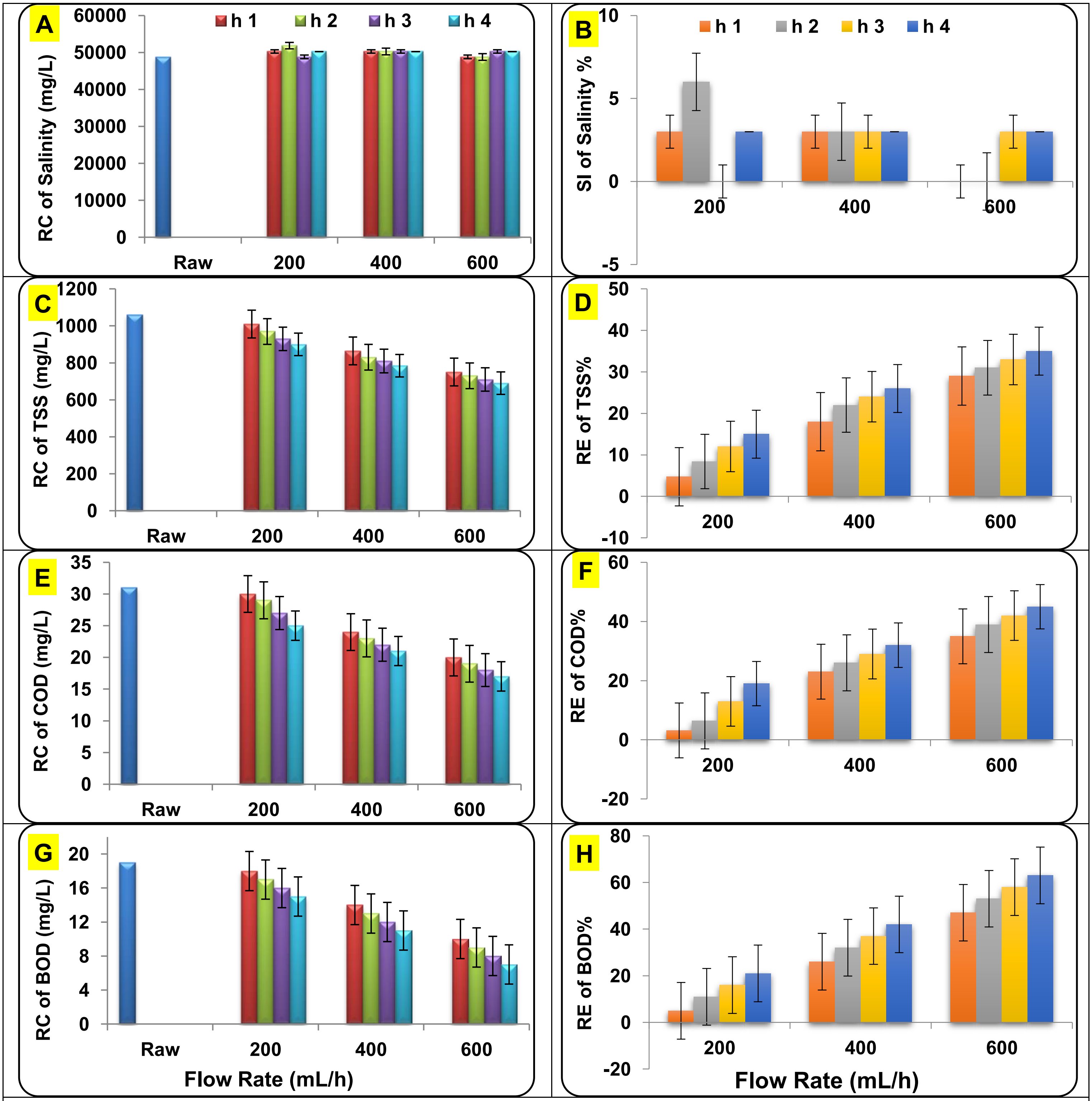

Figure S12. Residual Concentration (RC) and Removal/Increase % (RE/I%) of Salinity (A & B), TSS (C & D), COD (E & F) and BOD (G & H) after Continuous Treatment Using AgNPs/AC-NC Modified Cellulose Membrane/ Gravel Filter System at Different Flow Rates and Running Times.
